# Complement C3a Suppresses Spinal Cord Neural Stem Cell Activation by Inhibiting UCHL1 *via* the NF-κB p65/Nrf2 Pathway

**DOI:** 10.1007/s12264-025-01488-z

**Published:** 2025-10-03

**Authors:** Lu Ding, Xinyue Li, YaQin Guo, Feng-Quan Zhou, David Y. B. Deng

**Affiliations:** 1 Scientific Research Center, The Seventh Affiliated Hospital, Sun Yat-sen University, Shenzhen, 518107 China; 2 School of Medicine, Sun Yat-sen University, Shenzhen, 518107 China; 3 Sir Run Run Shaw Hospital, Zhejiang University School of Medicine, Hangzhou, 310016 China

**Keywords:** Complement C3a, Neural stem cell activation, UCHL1, NF-κB p65/Nrf2 pathway, Protein aggregation clearance, Spinal cord injury

## Abstract

Activation of spinal cord neural stem cells (NSCs) and subsequent neurogenesis holds a promising alternative for spinal cord injury (SCI) repair. Our previous study demonstrated that complement C3a, derived from reactive astrocytes, inhibits NSC proliferation by suppressing protein aggregate clearance through the deubiquitinating enzyme ubiquitin carboxy-terminal hydrolase L1 (UCHL1)-proteasome system post-SCI. However, the potential molecular mechanism by which C3a modulates NSC activation *via* this pathway remains unclear. Here, we revealed that C3a/C3a receptor (C3aR) signaling activated NF-κB p65, which in turn inhibited Nrf2 activity and UCHL1 expression, resulting in diminished proteasome activity and the accumulation of protein aggregates, and ultimately impaired NSC activation. Both knockdown of NF-κB p65 and Nrf2 upregulation restored UCHL1 expression and proteasome activity *in vitro*, promoting NSC activation by enhancing protein aggregate clearance. Mechanistically, we found that NF-κB p65 regulated Nrf2 activity through a dual mechanism: (1) promoting Keap1-dependent ubiquitination and proteasome degradation of Nrf2; (2) inhibiting protein kinase C-mediated Nrf2 phosphorylation and nuclear translocation. Using the dual-luciferase reporter assay and chromatin immunoprecipitation (ChIP) analysis, we further identified UCHL1 as a direct transcriptional target of Nrf2. Importantly, *in vivo* experiments using SCI mice confirmed that either C3aR blockade, NF-κB p65 knockdown, or Nrf2 overexpression could rescue SCI-induced UCHL1 downregulation. Together, this study uncovers the C3a-NF-κB p65-Nrf2-UCHL1-proteasome axis as a critical regulator of NSC activation after SCI. This may provide novel molecular targets and intervention strategies for SCI repair.

**Supplementary Information:**

The online version contains supplementary material available at 10.1007/s12264-025-01488-z.

## Introduction

Traumatic spinal cord injury (SCI) is a leading cause of lifelong neurological disability, which imposes a high psychological and financial burden on an individual’s life [1]. Despite the considerable advances in medical care [2], SCI poses a global health issue due to the limited supportive interventions without optimistic clinical efficacy. Stem cell therapy is promising for SCI treatment, among which modulating the endogenous neural stem cells (NSCs) to repair SCI is emerging as an optimistic alternative to cell transplantation. Accumulating evidence has demonstrated the latent potential of resident NSCs to repair SCI and brain injury [3–7].

Protein homeostasis is critically important for stem cell maintenance, proliferation, and differentiation. Recent studies have reported that increased protein aggregates reduce the ability of quiescent NSCs to activate, while the enhanced clearance of protein aggregates in NSCs mediated by the lysosome pathway and the proteasome ameliorates their capacity to exit quiescence, leading to NSC proliferation and neurogenesis *in vivo* [8, 9]. Interestingly, in our previous studies, we found that ubiquitin carboxy-terminal hydrolase L1 (UCH-L1), an indispensable deubiquitinating enzyme in the ubiquitin-proteasome system (UPS), serves as a key regulator of NSC activation, which promotes NSC proliferation after SCI by facilitating the clearance of protein aggregations *via* the proteasome; furthermore, complement C3a released from the reactive astrocytes post-SCI suppresses spinal cord NSC activation by inhibiting UCH-L1-proteasome functions, and blockade of C3a receptor (C3aR) enhances NSC proliferation and functional improvement after SCI [10]. However, the potential molecular mechanism by which C3a inhibits UCH-L1 to suppress NSC activation remains unknown.

The complement system, an important contributor to innate and adaptive immune responses, is rapidly activated and involved in immune inflammation after injury. C3 is the center of the three activation pathways of complement (classic, lectin, and alternative), and is cleaved into the bioactive fragments, C3a and C3b, upon activation by C3-convertase [11]. C3a plays a vital role in mediating neuroinflammation and is closely involved in the pathological progression of central nervous system (CNS) injuries and several neurodegenerative disorders [12]. The C3a/C3aR pathway activates disease-associated microglia and neurotoxic astrocyte signatures, as well as promoting lesion-related neuronal damage and white matter injury, thus contributing to neuroinflammation and neurological deficits [13–15]. C3 deficiency can reduce the secondary injury and promote neural regeneration and functional recovery after SCI by ameliorating inflammation [16]. Accumulating evidence suggests that C3-targeted intervention may hold promise as a viable therapy for CNS diseases.

Importantly, C3 plays a pivotal role in coordinating crosstalk with multiple immune and inflammatory networks. The transcription factor nuclear factor-kappa B (NF-κB) is a key regulator of inflammation in the peripheral system and CNS. NF-κB is composed of homo- and heterodimeric complexes of Rel family proteins, among which the NF-κB p65/p50 heterodimer is one of the best-studied and most abundant components. NF-κB p65/p50 is normally sequestrated in the cytoplasm by its inhibitory protein IκB. When stimulated by diverse extracellular stimuli such as pro-inflammatory factors and oxidative stress, the IκB degrades, then NF-κB p65/p50 is translocated to the nucleus and promotes the expression of target genes [17]. Aberrant NF-κB p65κ activation is tightly implicated in neural survival, synaptic plasticity, inflammation-induced nerve damage, and neurodegeneration in CNS diseases. Studies indicate that there are close collaborations between C3a and NF-κB p65. C3 can effectively activate NF-κB p65 to promote the release of large amounts of pro-inflammatory factors [18, 19]. In return, activation of NF-κB p65 in astrocytes facilitates the release of C3, resulting in impaired dendritic structure and neural network function by interacting with neuronal C3a receptors [15]. However, it remains unclear whether C3a inhibits UCH-L1 in spinal cord NSCs by NF-κB p65 activation.

Tissue cells are usually exposed to frequent changes of inflammation and oxidative stress after injury. Oxidative stress can cause mitochondrial dysfunction, protein misfolding, and DNA repair system damage by releasing large amounts of reactive oxygen species (ROS) and other free radicals [20]. Nuclear transcription factor erythroid-2 related factor 2 (Nrf2), a key regulator of anti-oxidative stress, plays critical roles in cell survival, proliferation, and DNA repair. Upon oxidative stress stimulation, Nrf2 is activated and transported to the nucleus, binds to the antioxidant response elements (AREs), and enhances the expression of downstream cytoprotective genes, such as those encoding antioxidant proteins, anti-inflammatory factors, and metabolic enzymes. [21]. Accumulating evidence has demonstrated complex functional crosstalk between the NF-κB p65 and Nrf2 pathways. Deficiency of Nrf2 enhances NF-κB p65 activity, leading to the production of inflammatory mediators, while NF-κB p65 can modulate the transcription and activity of Nrf2, thereby influencing the expression of Nrf2 target genes [22, 23]. Studies report that NF-κB p65 inhibits the Nrf2-ARE (Antioxidant Response Element) pathway either by interacting with Keap1 [24] or by removing CREB-binding protein (CBP) from Nrf2 [25].

As the master regulator against oxidative stress and inflammation, Nrf2 also exerts vital effects on NSC self-renewal and fate determination. Downregulation of Nrf2 is correlated with the impaired regenerative ability of neural stem/progenitor cells in the subventricular zone [26]. During NSC development, mitochondrial dynamics regulate their self-renewal and fate determination through Nrf2-dependent retrograde signaling [27]. Nrf2 enhances endogenous NSC proliferation, neural differentiation, and neurogenesis by reducing the ROS level [28]. Moreover, given that oxidative stress is a major driver of protein misfolding and protein aggregate accumulation, Nrf2 is intimately associated with protein homeostasis. It directly promotes the transcription of many proteasome-related genes (e.g., proteasome maturation protein) and key autophagy-associated genes, thereby facilitating the clearance of abnormal protein aggregates [29, 30]. Given the pivotal roles of Nrf2 in NSCs, we hypothesized that it may directly regulate UCH-L1 expression to modulate NSC activation. Nevertheless, whether C3a influences UCH-L1-mediated NSC activation *via* the NF-κB p65/Nrf2 pathway remains unknown.

In this study, we investigated the molecular mechanism by which C3a suppresses spinal cord NSC activation by UCH-L1. Our results reveal that C3a/C3aR signaling impairs proteasome activity and promotes protein aggregate accumulation through the NF-κB p65/Nrf2/UCH-L1 pathway, ultimately hindering quiescent NSC activation. Mechanistically, C3a-activated NF-κB P65 suppresses Nrf2 activity through two distinct means: (1) promoting the Keap1-dependent ubiquitination and degradation of Nrf2; (2) inhibiting Nrf2 phosphorylation and nuclear translocation. Furthermore, we identified UCH-L1 as a direct transcriptional target of Nrf2, with Nrf2 binding to the UCH-L1 promoter region to modulate its expression. These findings reveal a novel C3a-NF-κB p65-Nrf2-UCH-L1-proteasome axis that regulates NSC activation and may provide potential targets for the manipulation of NSC activation to repair SCI.

## Materials and Methods

### Materials and Animal Ethics Statement

All materials and antibodies used in this study are listed in Table S1. The newborn C57BL/6 mice (1 day postnatal) and adult C57BL/6 mice (8–10 weeks; half of male and female) were purchased from Zhuhai BesTest Bio-Tech Co., Ltd. All experimental procedures using laboratory animals were conducted in accordance with the Guide for the Care and Use of Laboratory Animals (National Research Council, 1996) and approved by the Animal Care and Use Committee of Sun Yat-sen University (Approval No.: SYSU-IACUC-2021-000438).

### Isolation and Culture of Spinal Cord NSCs

NSCs were obtained from the spinal cord of newborn C57BL/6 mice (1 day postnatal) as illustrated in Fig. S1A. Briefly, each newborn mouse was sacrificed and sterilized with 75% medical alcohol, then the spinal cord was separated and soaked in pre-cooled Hanks balanced salt solution (HBSS; without Ca^2+^/Mg^2+^; Gibco, Thermo Scientific, Waltham, USA), and the spinal meninges and blood vessels were carefully stripped under a dissecting microscope. Subsequently, the spinal tissues were cut into pieces using spring scissors until no visible tissue mass remained. The cell suspension was next filtered through a 70 µm cell strainer and centrifuged at 1000 rpm for 5 min to collect the cell precipitate. The cell pellets were resuspended and cultured with DMEM/F-12 (Gibco, Thermo Scientific, Waltham, USA) supplemented with 2% B-27™ (50×; Gibco, Thermo Scientific, Waltham, USA), 20 ng/mL epidermal growth factor (EGF; Peprotech, New Jersey, USA), 20 ng/mL fibroblast growth factor (FGF; Peprotech, NJ, USA), and 1% penicillin/streptomycin (10,000 U/mL, Gibco, Life Technologies, Waltham, USA). NSCs were passaged every 3 days using Accutase (Gibco, Thermo Scientific, Waltham, USA), and cells from passages 2–5 were selected for further investigation. All cells were tested for *Mycoplasma* contamination every three months.

### Identification of Spinal Cord NSCs

For cell identification, the NSC neurospheres were plated on sterile glass coverslips pre-coated with 0.1% poly-L-lysine (Gibco, Thermo Scientific, Waltham, USA), and identified using the typical NSC markers Nestin (1:300; Abcam, Cambridge, UK), SOX2 (1:300; Abcam), and vimentin (1:300; Abcam) by immunofluorescent staining. Then, the multilineage differentiation potential of NSCs was further evaluated. In brief, NSCs were digested into single-cell suspensions with Accutase and cultured in Neurobasal medium (Gibco, Thermo Scientific, Waltham, USA) supplemented with 2% B-27™ for 7 days. The generated neurons, astrocytes, and oligodendrocytes were separately labeled by anti-MAP2 antibody (1:200; Abcam), anti-GFAP antibody (1:300; Cell Signaling Technology, Danvers, USA), and anti-CNPase antibody (1:200; Cell Signaling Technology).

### NSC Activation Assay

To assess NSC activation, cells were maintained in growth factor-depleted basal medium (DMEM/F-12 without growth factors EGF/FGF) prior to treatment. After incubation with C3a (Sino Biological, Beijing, China) for 24 h or MG-132 (10 µmol/L; MedChemExpress, Monmouth Junction, NJ, USA) for 4 h, the medium was changed to basic medium supplemented with EGF/FGF, and NSCs were allowed to proliferate for another 24 h. NSC activation was then evaluated by EdU assay. Cells were co-cultured with 10 µmol/L EdU overnight before fixation with 4% paraformaldehyde (PFA), followed by permeabilization with 0.5% Triton X-100, and stained using the Click-iT EdU assay kit (US Everbright Inc.) in accordance with the manufacturer’s instructions. The proportions of EdU^+^ NSCs were analyzed *via* Cytoflex LX or laser scanning confocal microscopy.

### Proteostat Detection Assay

The formation of protein aggregates in NSCs was detected using the Proteostat® Aggresome Detection kit (Enzo, USA). NSCs were plated on sterile glass coverslips pre-coated with 0.1% poly-L-lysine before fixation with 4% PFA, then permeabilized with 0.5% Triton X-100 on ice and incubated with Proteostat Aggresome Detection Reagent (1:2000) for 30 min at room temperature (RT) and protected from light. The nuclei were next counterstained with DAPI (1:5000; Thermo Scientific, USA), and protein aggregates were visualized under the confocal microscope.

### Proteasome Activity Assay

The proteasome activity of NSCs was evaluated using the cell-permeable fluorescent proteasome activity probe Me4BodipyFl-Ahx3Leu3VS (Boston Biochem, Cambridge, MA, USA). Cells were incubated with 5 µmol/L proteasome activity probe for 2 h, protected from light in a cell incubator, before collection, then washed twice with PBS. The proteasomes labeled with probes were observed and analyzed using the confocal microscope.

### Lentivirus Construction and Cell Infection

The Nrf2 overexpression lentivirus (OE-Nrf2-LV) and the negative control lentivirus (NC-LV), NF-κB p65 knockdown lentivirus (NF-κB p65-shRNA) and the corresponding negative control lentivirus (NC-shRNA) were constructed by Genechem Co., Ltd (Shanghai, China). Lentivirus designed to overexpress Nrf2 was cloned into the GV358 vector (Ubi-MCS-3FLAG-SV40-EGFP-IRES-puromycin), and NF-κB p65 interference lentivirus was constructed with the GV493 vector (Ubi-MCS-3FLAG-SV40-EGFP-IRES-puromycin). The lentivirus vectors were co-transferred into HEK293T cells with the packaging plasmids, and the overexpression or knockdown efficiency was assessed after 48 h by qPCR. The final lentiviral titer of OE-Nrf2-LV and NC-LV was 5×10^8^ TU/mL. The final lentiviral titer of NF-κB p65-shRNA and NC-shRNA was 3×10^9^ TU/mL.

For viral infection *in vitro*, NSCs were separately infected with OE-Nrf2-LV, NF-κB p65-shRNA, and the corresponding negative control lentivirus (at a multiplicity of infection of 20). At 8 h after infection, the medium was replaced with fresh basal medium (FBS-free) without penicillin/streptomycin. At 48 h post-infection, the expression of Nrf2 and NF-κB p65 in cells was confirmed by qRT-PCR and Western blot analysis.

### Spinal Cord Injury Model and Experimental Groups

A SCI model with complete transection at the 10th thoracic vertebra (T10) was constructed in adult C57BL/6 mice (8–10 weeks; half male and half female) (Fig. 8A). After anesthesia with 2% isoflurane, the mice were positioned prone, the skin was incised, then the fascia and muscles were separated to expose the T9–10 spinal segments. Through a T10 laminectomy, the spinal cord was completely transected using a sharp scalpel to establish the T10 transection SCI model. In the Sham group, the T10 spinal cord was exposed without causing any additional damage. The SCI mice were randomly divided into 7 groups treated separately as follows: SCI group (0.9% normal saline; intraperitoneal injection [i.p.]; *n* = 6); SCI+C3aRa group (SB290157, 10 mg/kg, i.p.; *n* = 6); SCI group (3 µL Matrigel, injection *in situ*; *n* = 6); SCI+NC-shRNA group (3×10^9^ TU/mL; *n* = 6); SCI+NF-κB-shRNA group (3×10^9^ TU/mL; *n* = 6); SCI+NC-LV group (5×10^8^ TU/mL; *n* = 6); and SCI+OE-Nrf2-LV group (5×10^8^ TU/mL; *n* = 6). The C3aR antagonist SB290157 (10 mg/kg) or 0.9% normal saline was injected i.p. into SCI or Sham mice daily for 7 days. Lentiviral suspension (3 µL; mixed 1:1 with Matrigel) was injected directly into the lesion epicenter using a microsyringe.

After surgery, the mice were maintained at ~37°C using a heating pad until full recovery from anesthesia. The mice were subjected to manual bladder evacuations twice a day until sacrifice. No postoperative infections or mortality occurred. All mice survived to the endpoint with stable wounds.

### Co-immunoprecipitation (Co-IP) Assay

The Co-IP assay was conducted using an Immunoprecipitation Kit with Protein A+G Magnetic Beads (Beyotime Biotechnology, Shanghai, China). Firstly, the protein A/G beads were washed and incubated with the anti-NF-κB p65 antibody (1:100; Cell Signaling Technology), anti-Keap1 antibody (1:100; Proteintech, Wuhan, China), or anti-Nrf2 antibody (1:100; Cell Signaling Technology) for 30 min in a rotary shaker at RT before cell harvest. NSCs were pre-treated with MG-132 (10 μmol/L) for 4 h, C3a (5 μg/mL) or/and JSH-23 (3 μmol/L) for 24 h. Then the cells were collected and lysed in lysis buffer containing protease inhibitor cocktail (1:100) on ice for 20 min. The lysates were centrifuged at 14,000×g for 10 min at 4°C, then the supernatants were collected and incubated with the protein A/G beads pre-conjugated with target-specific antibodies in a rotary shaker at 4°C overnight. The bead-antibody-protein complexes were next washed with ice-cold lysis buffer. Beads were resuspended in the loading buffer (1×) and denatured at 95°C for 10 min in a dry bath incubator. Finally, supernatants containing eluted proteins were collected using a magnetic separation rack, and samples were immediately processed for Western blot analysis. Normal rabbit immunoglobulin G (IgG) was used as a negative control. The binding efficiency between Keap1 and Nrf2 was quantified by normalizing the intensity of co-precipitated Nrf2 to Keap1 in the IP lysate, with input levels as reference. Ubiquitinated Nrf2 levels were calculated as the ratio of Ub signal to input Nrf2 in IP lysates, with background subtraction using IgG controls.

### Cell Viability Assay

NSCs were enzymatically dissociated into a single-cell suspension using Accutase and seeded in 96-well plates at a density of 5×10^3^ cells/well in complete growth medium. Cells were treated with the following kinase activators separately at the indicated concentrations (0, 1, 5, 10, 20, or 40 μmol/L) for 24 h: SC-10 (PKC activator), SC79 (AKT activator), Ro67-7476 (ERK1/2 activator), or GSK621 (AMPKα activator). Then 10 μL of CCK-8 solution was added to each well, and cells were incubated for another 2 h (protected from light) in an incubator. The absorbance at 450 nm was next measured using a microplate reader.

### Chromatin Immunoprecipitation (ChIP) Assay

The ChIP assay was performed using the Enzymatic ChIP Assay Kit (Beyotime Biotechnology) according to the instructions of the manufacturer. Briefly, NSCs were cross-linked with 1% PFA for 10 min at 37°C, then this process was terminated by glycine solution at RT. After washing with pre-cooled PBS, the cell pellets were incubated with MNase at 37°C for 20 min and terminated using EDTA. The pellets were resuspended in CHIP buffer and ultrasonicated to fragment the chromatin. Then the supernatants were collected after centrifugation and incubated overnight with anti-Nrf2 antibody (1:200; Cell Signaling Technology) in a rotary shaker at 4°C, and subsequently incubated with Protein A/G Magnetic Beads/Salmon Sperm DNA at 4°C for 1 h. The chromatin fragments conjugated with the beads were collected in Elution Buffer (1% SDS, 0.1 mol/L NaHCO_3_) and incubated with 5 mol/L NaCl at 65°C for 2 h to remove the cross-linking between proteins and genomic DNA. The DNA was further purified using a DNA purification kit (Beyotime Biotechnology). Normal rabbit IgG was included as a negative control. The bound DNA fragments were analyzed by real-time PCR using the specific primers as follows: *Uchl1* promoter, Forward (5'–3'): TAGGGGTTTGGGAGTGACTG, Reverse (5'–3'): CAAACACACACACACGGGTC.

### Dual-luciferase Reporter Assay

The Nrf2 overexpression plasmid pcDNA3.1(+)-V5-Nfe2l2 (m-Nrf2) and negative control plasmid pcDNA3.1(+)-V5, the Firefly luciferase reporter plasmid with the *Uchl1* promoter sequences pGL4.19-mUchl1 promoter (−2000 to +100) (m-UCH-L1 [full]) and the corresponding luciferase reporter plasmids with *Uchl1* promoter sequence mutations m-UCH-L1 (Mut1/Mut2/Mut3), the *Renilla* luciferase control plasmids were all constructed by the Public Protein/Plasmid Library (Jiangsu, China). HEK293T cells were obtained from the American Type Culture Collection. Prior to experiments, HEK293T cells were authenticated by short tandem repeat profiling and were routinely tested for *Mycoplasma* contamination every three months.

For the dual-luciferase reporter assay, HEK293T cells were co-transfected with m-Nrf2, m-UCHL1 (full), or its mutant plasmids m-UCHL1 (Mut1/Mut2/ Mut3) and the *Renilla* luciferase control plasmid using the liposomal transfection reagent (Yeasen, Shanghai, China). After transfection for 48 h, cells were lysed with the lysis buffer for 30 min at RT. Then the luciferase assay buffer was added to the cell lysate, and the Firefly luciferase activity was immediately measured using a multimode microplate reader. The reaction was subsequently terminated, and the *Renilla* luciferase activity was measured. The relative luciferase activity was quantified as the ratio of Firefly luciferase activity/*Renilla* luciferase activity.

### Immunofluorescent Staining

Following C3a or JSH-23 treatment, NSCs were plated on sterile glass coverslips pre-coated with 0.1% poly-L-lysine, fixed in 4% PFA for 20 min, and permeabilized in 0.3% Triton X-100 for 30 min. Then the cells were blocked with 5% BSA for 1 h at RT and incubated with primary antibodies overnight at 4°C, subsequently incubated with Alexa Fluor 488/647-conjugated secondary antibodies for 1.5 h at RT (1:500; Thermo Scientific, Waltham, USA), and counterstained with DAPI (Sigma, St. Louis. MO, USA). All immunofluorescence images were acquired using a Zeiss LSM 880 confocal microscope. The primary antibodies used in this study were as follows: Nestin (1:300; Abcam, Cambridge, UK), SOX2 (1:300; Abcam), vimentin (1:300; Abcam), MAP2 (1:200; Abcam), GFAP (1:300; Cell Signaling Technology, CST, Danvers, USA), CNPase (1:200; Cell Signaling Technology), and NF-κB p65 (1:300; Cell Signaling Technology).

### Quantification of Confocal Analysis

All confocal images were examined using a Zeiss LSM 880 confocal microscope and quantified in a blinded manner. To evaluate NSC activation, the proportion of EdU-positive cells relative to total DAPI-stained nuclei was quantified among different groups. To quantify the aggregate formation and proteasome activity in NSCs, images with similar numbers of cells were randomly selected, and the relative fluorescence area of protein aggresomes (red) or proteasomes labeled by probes (green) in single cells was quantified across different treatments. The NF-κB p65 nuclear translocation was quantified as the ratio of the overlapping area of green fluorescence and blue fluorescence (NF-κB p65/DAPI^+^) relative to the total area of blue fluorescence (DAPI^+^). Each data point represents one biological repeated experiment. All experiments were conducted with at least three biological replicates.

### Quantitative Real-time Polymerase Chain Reaction (qRT-PCR)

Total RNA from NSCs was extracted using the Universal RNA extraction kit (Accurate Biology, Hunan, China) according to the manufacturer’s instructions. The RNA concentration and purity were determined by Nanodrop One (ThermoFisher, USA). 0.4 μg of total RNA was used for cDNA synthesis by reverse transcription using the PrimeScript RT reagent kit (Accurate Biology), and a qRT-PCR assay was conducted with the SYBR Premix EX Taq (Accurate Biology). The PCR results were recorded as threshold cycle (Ct) numbers and normalized to those of glyceraldehyde 3-phosphate dehydrogenase (GAPDH). The relative expression of genes was calculated as fold-change using the 2^-△△Ct^ method. GAPDH was used as the internal control. The primer sequences for genes used here are listed in Table S2.

### Extraction of the Nuclear Proteins and Total Cell Proteins

The nuclear proteins from NSCs were extracted using the Nuclear Protein and Cytoplasmic Protein Extraction Kit (Beyotime Biotechnology, Shanghai, China) in accordance with the instructions provided by the manufacturer. Briefly, cells were first lysed on ice for 10 min in the cytoplasmic protein extraction reagent A supplemented with PMSF, then the cytoplasmic protein extraction reagent B was added. The lysates were next centrifuged at 12000 g for 5 min at 4°C, and the cell suspensions were removed as much as possible to obtain the nuclear precipitates. Then the cell precipitates were incubated with nuclear protein extraction reagent supplemented with PMSF on ice for 30 min, with high speed and intense vortexing for 15–30 s every 1–2 min. Finally, the nuclear lysate was centrifuged at 12000 g for 10 min at 4°C, and the cell suspension was collected as the nuclear proteins. All samples were subsequently denatured for Western blotting analysis.

To extract the total cell proteins, cells and the harvested injured spinal tissues were lysed in RIPA buffer (Epizyme Biomedical Technology, Shanghai, China) containing PMSF and protease inhibitor cocktails (Epizyme Biomedical Technology, Shanghai, China) on ice for 30 min, followed by centrifugation at 12000 rpm for 15 min at 4°C. The cell suspensions were then collected as the total cell proteins and subsequently denatured for Western blotting analysis.

### Western Blotting Analysis

A total of 20 µg of cell proteins were separated on a 10% sodium dodecyl sulfate-polyacrylamide electrophoresis (SDS-PAGE) gel and transferred to polyvinylidene difluoride membranes (Millipore, Mississauga, Canada). Then the membranes were blocked with 5% non-fat milk and incubated with the primary antibodies overnight at 4°C with gentle shaking, followed by combination with horseradish peroxidase-conjugated secondary antibodies for 1 h at RT. The protein enrichment was visualized using an enhanced chemiluminescence reagent in a chemiluminescence image analysis system (Bio-Rad, USA), and the grayscale analysis of bands was determined by ImageJ. The primary antibodies applied were as follows: C3aR (1:500; Santa Cruz, CA, USA), NF-κB p65 (1:1000; Servicebio Technology, Wuhan, China), phospho-NF-κB p65 (p-NF-κB p65; 1:1000; Servicebio Technology), Nrf2 (1:1000; Servicebio Technology), phospho-Nrf2 (p-Nrf2; 1:1000; Proteintech, Wuhan, China), UCHL1 (1:1000; Cell Signaling Technology), Keap1 (1:1000; Servicebio Technology), GAPDH (1:3000; Proteintech), β-actin (1:1000; Servicebio Technology), Histone H3 (1:1000; Proteintech).

### Statistical Analysis

Each experimental procedure was performed at least three times independently. Statistical analysis was applied using SPSS 20.0 software (SPSS Inc., Chicago, IL, USA) and GraphPad Prism 7. Two-tailed unpaired Student’s *t*-test was used for comparisons between two groups, and one-way analysis of variance (ANOVA) with Bonferroni *post hoc* analysis was applied to multiple comparisons. All data are presented as the mean ± standard error of the mean (SEM) with *n* = 3 per group. *P* < 0.05 was considered to be statistically significant (*P < 0.05; **P < 0.01; ***P < 0.001).

## Results

### C3a Inhibits Spinal Cord NSC Activation by Suppressing Protein Aggregate Clearance *via* the Ubiquitin-Proteasome System

We have previously revealed that C3a inhibits the proliferation of fetal brain-deprived NSCs by downregulating UCH-L1 *in vitro* [10]. To further investigate the effects of C3a on spinal cord NSC activation, NSCs from newborn mouse spinal cord were isolated (Fig. S1A), and their stem cell properties were evaluated. NSCs exhibited robust self-renewal as evidenced by the formation of neurospheres with typical shape and refractive index (Fig. S1B). Both neurospheres and free single NSCs were immunopositive for the NSC markers Nestin, Sox2, and vimentin (Fig. S1B). Notably, these cells maintained multipotent differentiation potential comparable to brain-derived NSCs, which could differentiate into the three typical neural cell lines: neurons (MAP2^+^), astrocytes (GFAP^+^), and oligodendrocytes (CNPase^+^) when cultured in differentiation medium (Fig. S1C). These data indicate that the spinal cord NSCs show robust stemness characteristics *in vitro*.

**Fig. 1:**
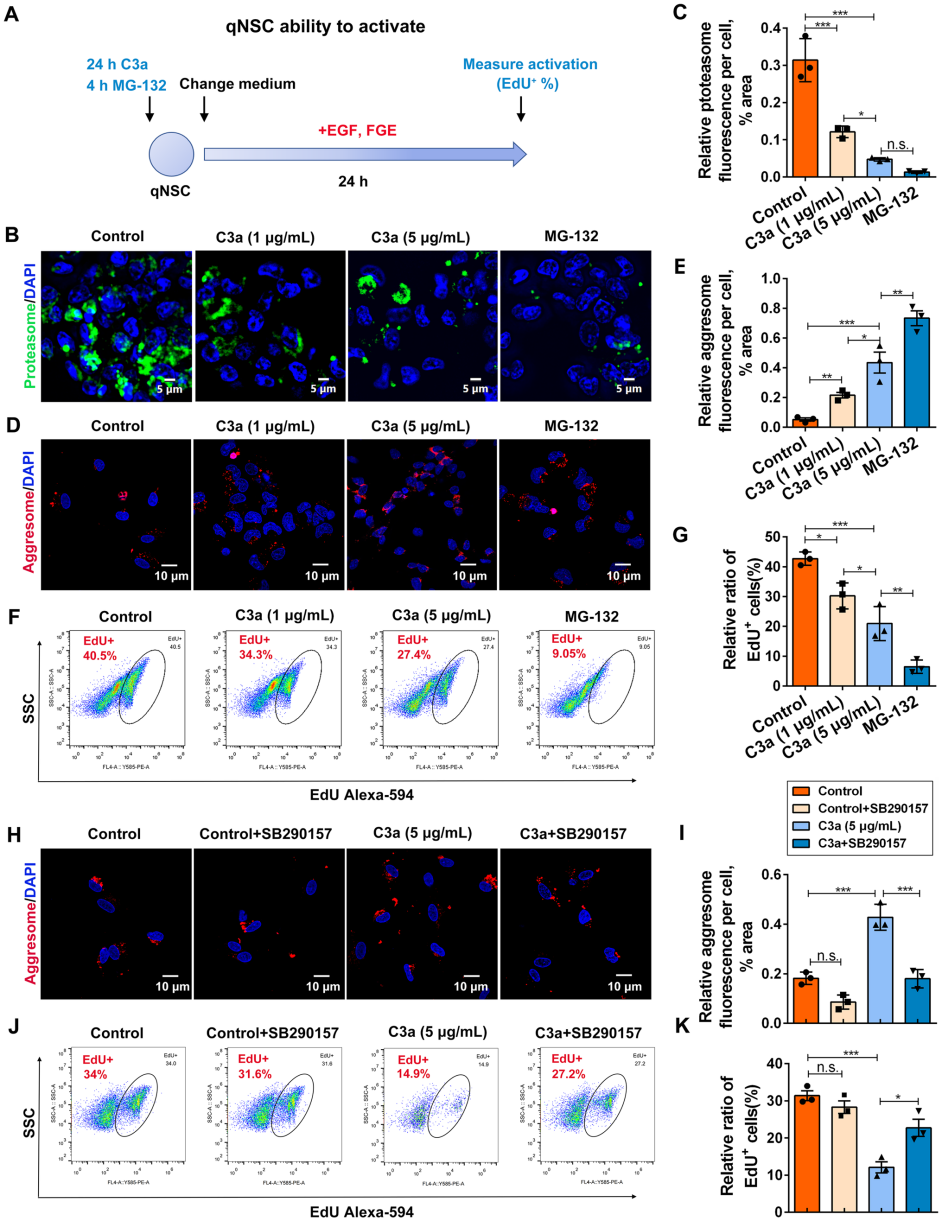
C3a inhibits spinal cord NSC activation by suppressing protein aggregate clearance mediated by the ubiquitin-proteasome system. **A** Diagram showing the process of spinal cord quiescent NSC (qNSC) activation in response to growth factors. Following treatment with C3a for 24 h or MG132 for 4 h, the basal medium (free of EGF/FGF) is removed, and NSCs are cultured in the proliferative medium supplemented with growth factors EGF and FGF for another 24 h. The activity of NSCs is evaluated by the EdU (cells in S phase of the cell cycle) incorporation assay. **B** Representative immunofluorescence images of NSCs stained with a proteasome probe (proteasomes, green) after treatment with C3a or MG-132. Scale bars, 5 μm. **C** Quantification of proteasome activity measured by proteasome probes. *n* = 3 biological replicates. **D** Representative immunofluorescence images of NSCs stained with Proteostat (protein aggregates, red) after treatment with C3a or MG-132. Scale bars, 10 μm. **E** Quantification of Proteostat fluorescence in NSCs treated with C3a or MG-132. *n* = 3 biological replicates. **F** NSC activation measured using EdU incorporation assay detected by intracellular flow cytometry. **G** The percentages of EdU^+^ NSCs measured by intracellular flow cytometry. *n* = 3 biological replicates. **H, I** Representative immunofluorescence images and quantification of protein aggregates stained with Proteostat in NSCs treated with C3a or C3a+C3aR antagonist SB290157. Scale bars (**H**), 10 μm. (**I**) *n* = 3 biological replicates. **J, K** Representative images and quantification of EdU^+^ NSCs treated with C3a or C3a+C3aR antagonist SB290157 by intracellular flow cytometry. (**K**) *n* = 3 biological replicates. Data are presented as the mean ± SEM. *P < 0.05, **P < 0.01, ***P < 0.001, n.s. not significantly different, by one-way ANOVA with Bonferroni *post hoc* analysis.

Quiescent NSCs can activate and generate active NSCs in response to growth factor signals. To evaluate whether C3a affects spinal cord NSC activation through UPS-associated protein aggresome clearance, the NSC activation assay was applied as previously described [9] (Fig. 1A), instead of evaluating NSC proliferation directly. NSCs were treated with MG-132 for 4 h or C3a for 24 h in the basal medium without growth factors EGF/FGF before changing to growth medium supplemented with EGF/FGF (Fig. 1A). MG-132, an inhibitor of proteasome activity, markedly inhibited proteasome activity (Fig. 1B, C) and protein aggregate clearance (Fig. 1D, E), thus suppressing NSC activation (Fig. 1F, G). Similarly, treatment with C3a resulted in impaired proteasome activity (Fig. 1B, C) and increased accumulation of protein aggregates (Fig. 1D, E), causing an impaired capacity of NSCs to be activated in response to growth factors (Fig. 1F, G). Furthermore, the blockade of C3a signaling using the C3aR antagonist SB290157 led to less accumulation of protein aggregates (Fig. 1H, I) and enhanced NSC activation (Fig. 1J, K). These results revealed that C3a inhibits spinal cord NSC activation through UPS-mediated protein aggresome clearance.

### C3a might Suppress Spinal Cord NSC Activation by Inhibiting UCHL1 through the NF-κB p65/Nrf2 Pathway

**Fig. 2:**
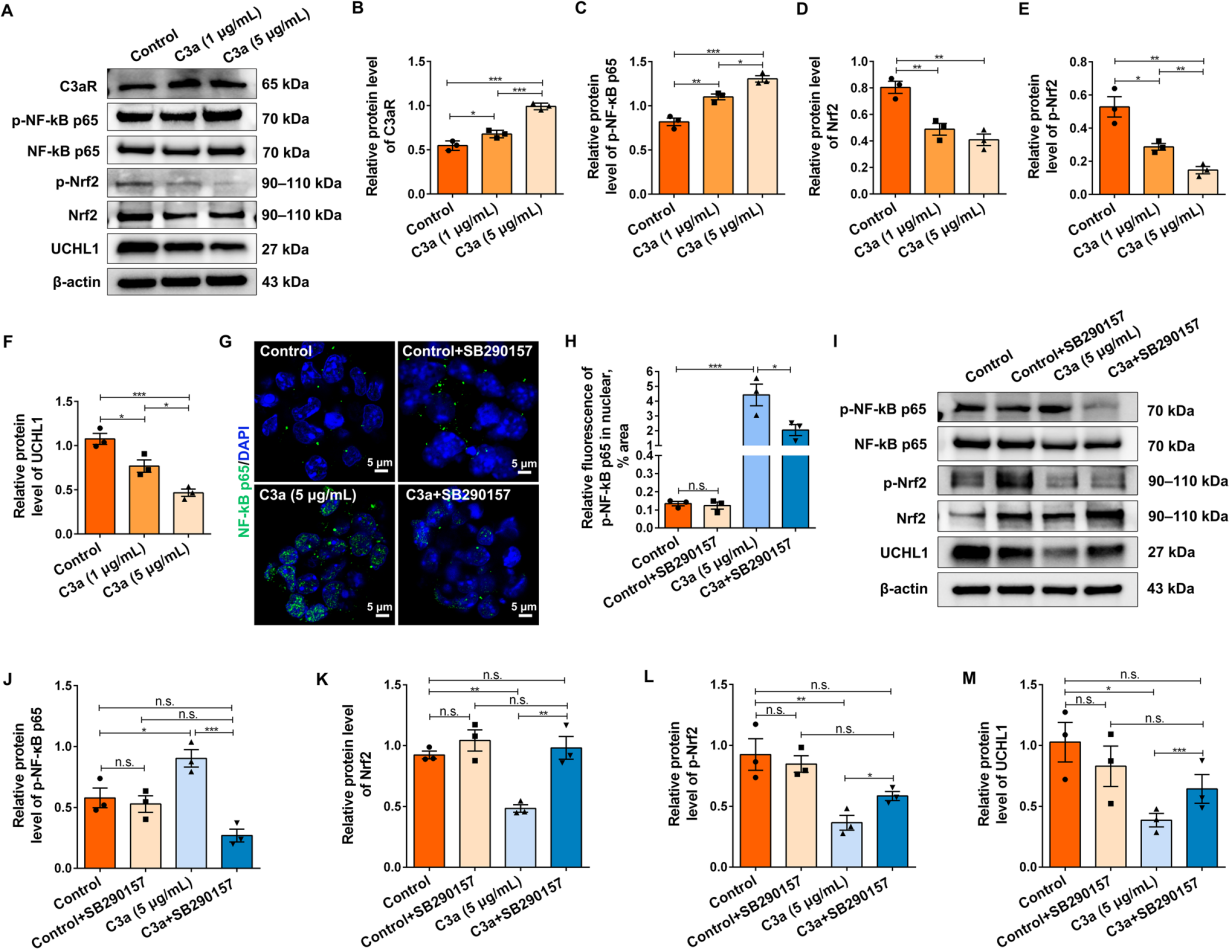
C3a suppresses spinal cord NSC activation by inhibiting UCHL1 *via* the NF-κB p65/Nrf2 pathway. **A** NSCs treated with DMSO and C3a for 24 h, and the cell lysates were analyzed by Western blotting with specific antibodies. **B–F** Semiquantitative results of the blots in **A**. Relative levels of p-NF-κB p65 normalized to NF-κB p65. Relative levels of p-Nrf2 normalized to Nrf2. Relative levels of C3aR, Nrf2, and UCHL1 normalized to β-actin. *n* = 3 biological replicates. **G** Immunofluorescence staining of NF-κB p65 (green) in NSCs treated with DMSO, C3a, or along with C3aR antagonist SB290157 for 24 h. Scale bars, 5 μm. **H** Quantification of NF-κB p65 translocated into the nucleus. *n* = 3 biological replicates. **I–M** Western blots and quantification of p-NF-κB p65, NF-κB p65, p-Nrf2, Nrf2, and UCHL1 in NSCs treated with DMSO, C3a, or along with C3aR antagonist SB290157 for 24 h. Relative levels of p-NF-κB p65 normalized to NF-κB p65. Relative levels of p-Nrf2 normalized to Nrf2. Relative levels of Nrf2 and UCHL1 normalized to β-actin. (**J–M**) *n* = 3 biological replicates. Data are presented as the mean ± SEM. *P < 0.05, **P < 0.01, ***P < 0.001, n.s. not significantly different, by one-way ANOVA with Bonferroni *post hoc* analysis.

The complement system functions to coordinate multiple immune and inflammatory pathways to modulate the progression of CNS diseases. Evidence has revealed that C3 plays critical roles in several inflammatory disorders by NF-κB p65 signaling activation [18, 19]. To evaluate whether C3a inhibits NSC activation *via* the NF-kB p65 pathway, spinal cord NSCs were treated with C3a (at 1 and 5 μg/mL). The results showed that C3a administration promoted the expression of C3aRs and p-NF-κB p65 in a concentration-dependent manner (Fig. 2A–C). Oxidative stress and an inflammatory microenvironment are the two important mechanisms responsible for NSC activation and fate determination after SCI. Nrf2 and NF-κB p65 function as the critical transcription factors in response to oxidative stress and inflammation, respectively. A large amount of evidence has demonstrated that there is complicated functional crosstalk between the Nrf2 and NF-κB p65 pathways [22, 31]. Consistent with this, the protein levels of Nrf2 and p-Nrf2, as well as UCH-L1, were significantly downregulated in the C3a treatment group during NSC activation (Fig. 2A, D–F). Given that treatment with 5 μg/mL of C3a led to a more significant biological effect in NSCs, it was applied as the experimental condition in the subsequent experiments. Immunofluorescence staining showed that C3a significantly facilitated the nuclear translocation of NF-κB p65 in NSCs, which was reversibly blocked by the C3aR antagonist SB290157 (Fig. 2G, H). Moreover, SB290157 markedly inhibited the upregulation of p-NF-κB p65 resulting from C3a treatment (Fig. 2I, J), while enhancing the expression of Nrf2, p-Nrf2, and UCH-L1 (Fig. 2I, K–M). Our results suggested that C3a can inhibit spinal cord NSC activation through the NF-κB p65/Nrf2/UCH-L1 pathway.

### NF-κB p65 Impedes NSC Activation Through Inhibiting Nrf2/UCH-L1 Signaling

**Fig. 3:**
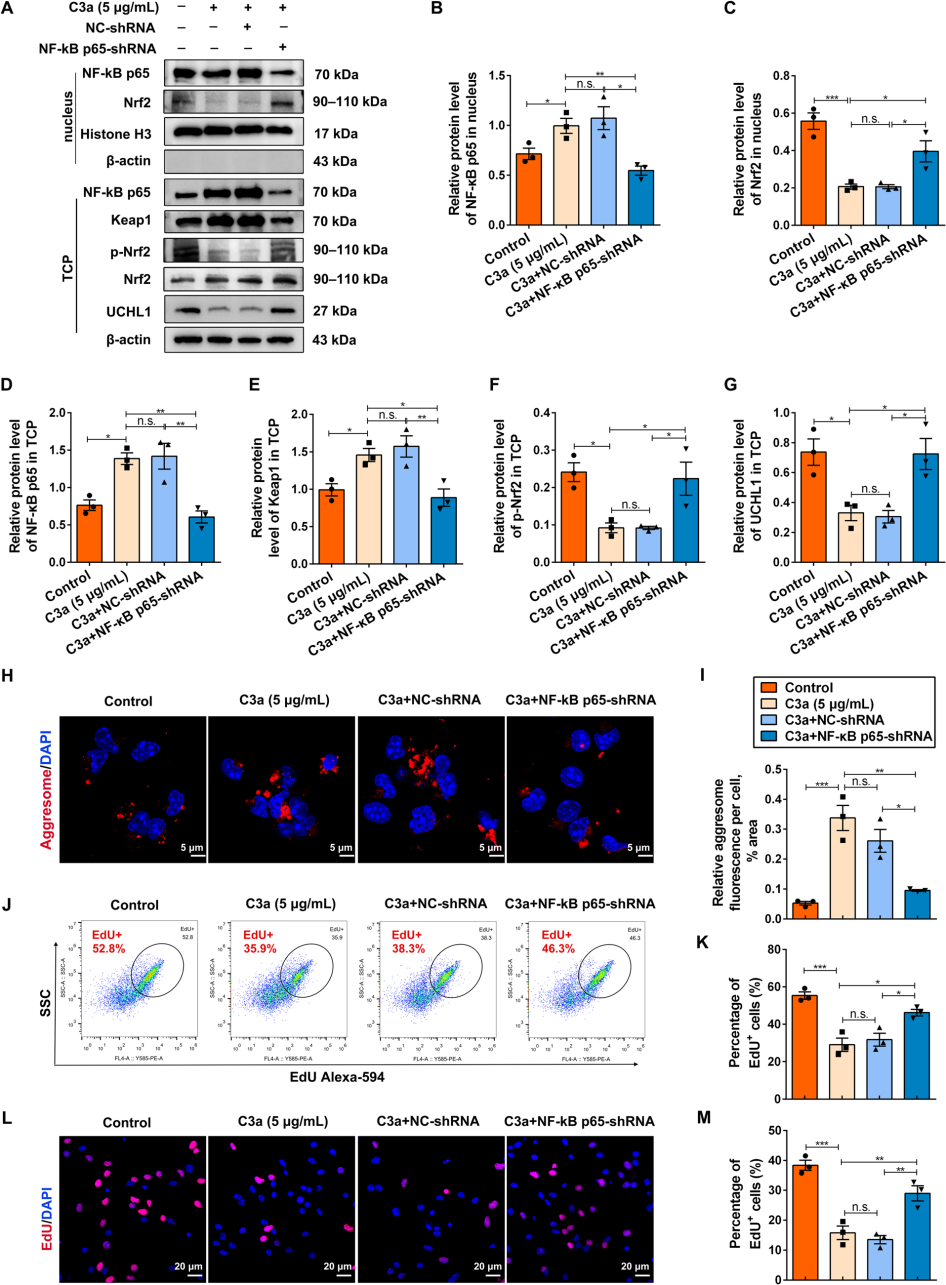
Knockdown of NF-κB p65 facilitates NSC activation through upregulation of Nrf2/UCHL1 signaling. **A** NSCs are treated with DMSO, C3a, or along with negative control lentivirus (NC-shRNA) and NF-κB p65 knockdown lentivirus (NF-κB p65-shRNA), then the nuclear protein and total cell protein (TCP) are separately extracted. The nuclear proteins are immunoblotted with anti-NF-κB p65, anti-Nrf2, anti-Histone H3, and anti-β-actin. TCP is probed by immunoblotting by NF-κB p65, Keap1, p-Nrf2, Nrf2, UCHL1, and β-actin. **B–G** Semiquantitative results of the blots in **A**. The protein levels of nuclear NF-κB p65 and nuclear Nrf2 are normalized to Histone H3. Relative levels of p-Nrf2 in TCP normalized to Nrf2. Relative levels of NF-κB p65, Keap1, and UCHL1 in TCP normalized to β-actin. Histone H3 and β-actin serve as the internal controls. *n* = 3 biological replicates. **H, I** The aggregated proteins in NSCs treated with DMSO, C3a, or along with NC-shRNA and NF-κB p65-shRNA stained with Proteostat (red). Scale bars (**H**), 5 μm. (**I**) *n* = 3 biological replicates. **J, K** Following treatment with DMSO, C3a, or/with NC-shRNA and NF-κB p65-shRNA, NSC activation is evaluated using EdU incorporation assay by intracellular flow cytometry, and the percentage of EdU^+^ cells is quantified (**K**). *n* = 3 biological replicates. **L, M** NSCs subjected to DMSO, C3a, or/with NC-shRNA and NF-κB p65-shRNA treatment, immunostained for EdU (red). The relative percentages of EdU^+^ cells are quantified among different groups (**M**). Scale bars (**L**), 5 μm. (**M**) *n* = 3 biological replicates. Data are presented as the mean ± SEM. *P < 0.05, **P < 0.01, ***P < 0.001, n.s. no significant difference by one-way ANOVA with Bonferroni *post hoc* analysis.

NF-κB p65 regulates the expression of Nrf2 downstream target genes by modulating its transcriptional expression and activity. To further verify the regulation of Nrf2 and UCH-L1 by NF-κB p65 under C3a stimulation, NF-κB p65 knockdown lentivirus (NF-κB p65-shRNA) was constructed, and the knockdown effectiveness in NSCs was confirmed. Among the three NF-κB p65-shRNAs, NF-κB p65-shRNA-2 showed the best knockdown effect (Fig. S2A–C) and was selected for the subsequent experiments. NSCs were treated with DMSO, C3a, or with NF-κB p65-shRNA and NC-shRNA separately, and the activation of NF-κB p65 and Nrf2 was detected using the expression levels of their nuclear proteins and the phosphorylated protein in the total cell proteins (TCP). Consistently, C3a significantly activated NF-κB p65, evidenced by both the upregulation of NF-κB p65 in the nucleus and p-NF-κB p65 in the TCP (Fig. 3A, B, D). Moreover, C3a promoted the expression of Keap1 (a key negative regulator of Nrf2) (Fig. 3A, E), while simultaneously reducing the protein levels of nuclear Nrf2, p-Nrf2, and UCH-L1 in the TCP (Fig. 3A, C, F, G). In contrast, knockdown of NF-κB p65 significantly suppressed Keap1 expression, enhanced Nrf2 nuclear translocation, and increased the protein levels of p-Nrf2 and UCH-L1 (Fig. 3A–G), indicating that C3a inhibits Nrf2/UCH-L1 signaling by NF-κB p65 activation. The protein aggregate clearance and NSC activation were further evaluated. Compared to the C3a-treated group, NF-κB p65 knockdown reduced the accumulation of protein aggresomes (Fig. 3H, I) and enhanced NSC activation (Fig. 3J–M).

**Fig. 4:**
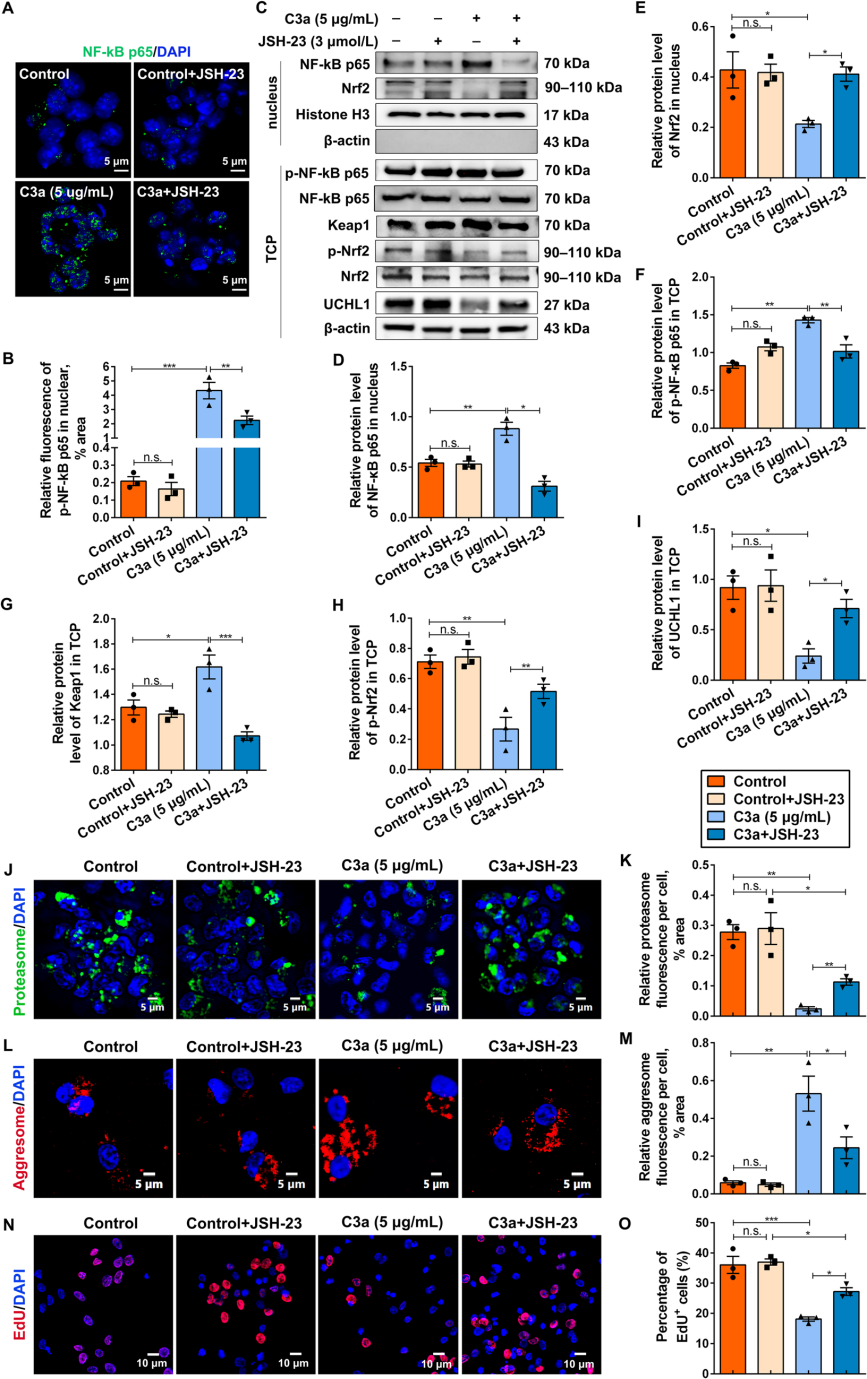
The NF-κB p65 inhibitor, JSH-23, enhances NSC activation by facilitating Nrf2 activation and UCHL1 expression. **A** NSCs treated with DMSO, C3a, or along with the NF-κB p65 inhibitor JSH-23 for 24 h and then immunostained for anti-NF-κB p65 (green) antibody. Scale bars, 5 μm. **B** Quantification of the NF-κB p65 translocated into the nucleus. **C–I** After stimulation with DMSO, C3a, or along with JSH-23 for 24 h, the nuclear proteins and TCP in NSCs are extracted and then probed by western blot for specific antibodies. **D–I** The semiquantitative results of the blots in **C**. The protein levels of nuclear NF-κB p65 and nuclear Nrf2 are normalized to Histone H3. Relative levels of p-NF-κB p65 and p-Nrf2 normalized to NF-κB p65. And Nrf2 in TCP. Relative levels of NF-κB p65, Keap1, and UCHL1 in TCP are normalized to β-actin. Histone H3 and β-actin serve as the internal controls. **J–K** Representative immunofluorescence images and quantification (**K**) of NSCs stained with proteasome probes (proteasome, green) after treatment with DMSO, C3a, or along with JSH-23 for 24 h. Scale bars (**J**), 5 μm. **L, M** Following treatment with DMSO, C3a, or JSH-23 for 24 h, NSCs are fixed and stained for aggregated proteins with Proteostat (red). Quantification is shown in L. Scale bars (**L**), 5 μm. **N, O** NSCs are treated with DMSO, C3a, or along with the NF-κB p65 inhibitor JSH-23 for 24 h. During the treatment, cells were incubated with EdU overnight, then fixed and analyzed for the percentage of EdU^+^ NSCs. Scale bars (**N**), 10 μm. (**B/D–I/K/M/O**) *n* = 3 biological replicates. Data are presented as the mean ± SEM. *P < 0.05, **P < 0.01, ***P < 0.001, n.s. no significant difference, by one-way ANOVA with Bonferroni *post hoc* analysis.

Next, JSH-23, an NF-κB p65 inhibitor serving to suppress its transcriptional activity, was further applied to validate these findings. Consistent with the results above, JSH-23 markedly suppressed the nuclear translocation of NF-κB p65 resulting from C3a stimulation (Fig. 4A, B), and decreased the protein levels of nuclear NF-κB p65 and Keap1 (Fig. 4C–F), accompanied by the upregulation of nuclear Nrf2, p-Nrf2, and UCH-L1 in NSCs (Fig. 4C, G–I). Similarly, treatment with C3a+JSH-23 restored the impaired proteasome activity (Fig. 4J, K), promoted aggregate clearance (Fig. 4L, M), and enhanced NSC activation compared to the C3a group (Fig. 4N, O). Taken together, our data provided unequivocal support for the conclusion that C3a-mediated NF-κB p65 activation inhibits NSC activation by suppressing the Nrf2/UCH-L1 signaling pathway.

### NF-κB p65 Inhibits Nrf2 by Facilitating the Keap1-Dependent Ubiquitination Degradation of Nrf2, and Blocking Its Phosphorylation and Nuclear Translocation

Accumulating evidence has demonstrated the crosstalk between NF-κB p65 and Nrf2. However, the molecular mechanism underlying the NF-κB p65 modulation of Nrf2 in a specific context is unknown. It has been reported that NF-κB p65 inhibits the Nrf2-ARE pathway through interaction with Keap1 [24], a well-known repressor of Nrf2. In the present study, C3a treatment led to the upregulation of NF-κB p65 and Keap1, whereas the protein level of Keap1 was decreased when NF-κB p65 was inhibited, suggesting that NF-κB p65, activated by C3a, promotes Keap1 expression (Figs 3A, 4B).

**Fig. 5:**
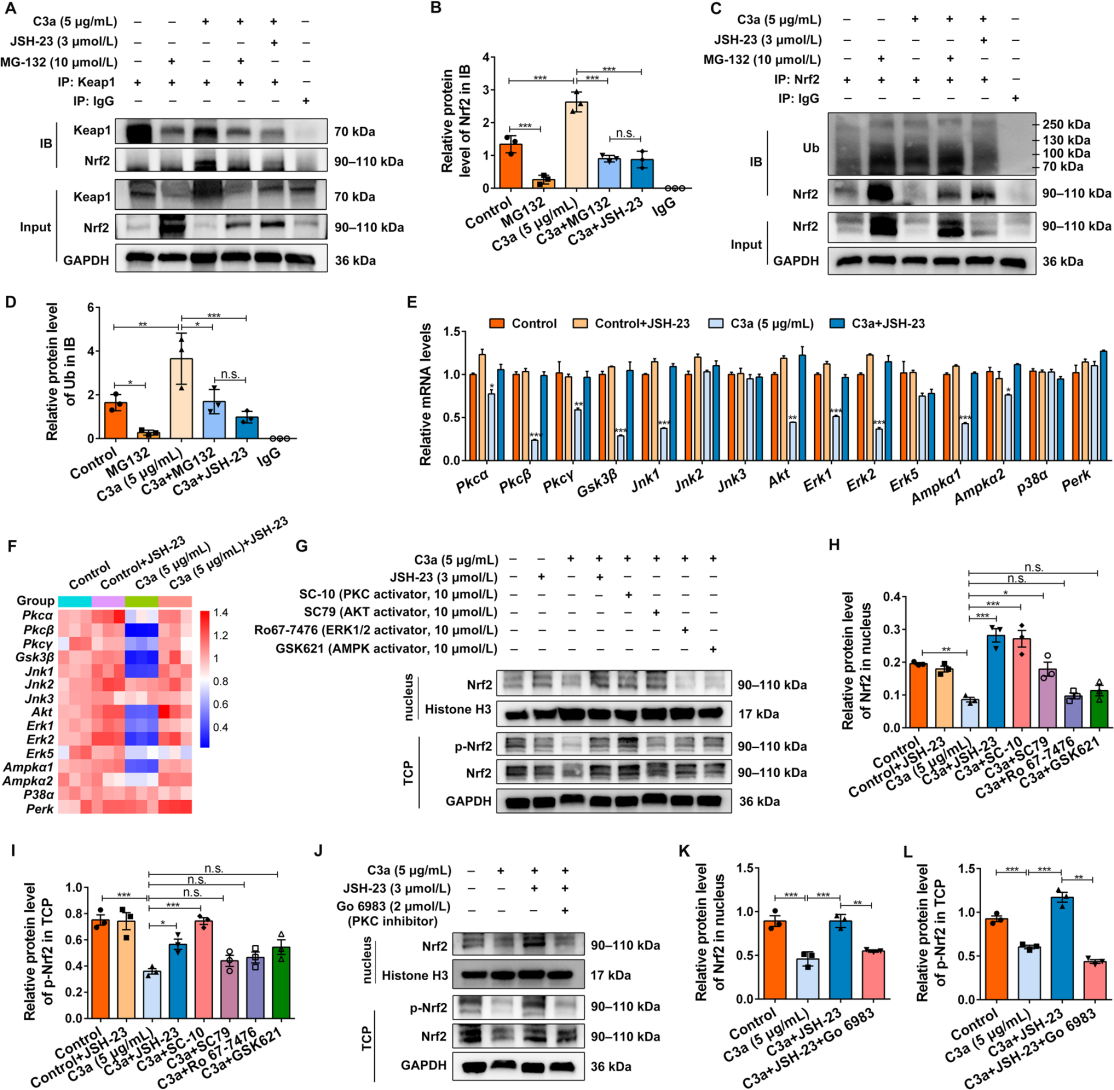
NF-κB p65 inhibits Nrf2 by enhancing the Keap1-dependent Nrf2 ubiquitylation degradation and suppressing Nrf2 phosphorylation mediated by PKC. **A** Whole NSC lysates were immunoprecipitated with anti-Keap1 antibody and analyzed by immunoblotting using antibodies against Keap1 and Nrf2. Input: 10% of the lysate; IgG as a negative control; GAPDH as an internal control. **B** Binding efficiency quantified by normalizing the intensity of co-precipitated Nrf2 to Keap1 in the IP lysate, with input levels as reference. *n* = 3 biological replicates. **C** Nrf2 ubiquitination detected by Co-IP using antibodies against Ub and Nrf2. IgG as a negative control; GAPDH as an internal control. **D** Ubiquitinated Nrf2 levels calculated as the ratio of Ub signal to input Nrf2 in IP lysates, with background subtraction using IgG controls. **E** The mRNA expression of protein kinases in NSCs treated with DMSO, C3a, or along with JSH-23 for 24 h was measured by qRT-PCR, normalized to GAPDH. *n* = 3 biological replicates. **F** Heat map of the relative expression of protein kinases among different groups. **G–I** NSCs treated with DMSO, C3a, or along with JSH-23, PKC activator SC-10, AKT activator SC79, ERK1/2 activator Ro67-7476, and AMPK activator GSK621 for 24 h, then the nuclear proteins and TCP are extracted separately. The relative expressions of nuclear Nrf2, p-Nrf2, and Nrf2 in TCP detected by Western blots (**G**) and quantified using ImageJ (**H, I**). The protein level of nuclear Nrf2 is normalized to Histone H3; the relative level of p-Nrf2 in TCP is normalized to Nrf2; Histone H3 and GAPDH serve as the internal control. (**H, I**) *n* = 3 biological replicates. **J–L** NSCs are treated with DMSO, C3a, or along with JSH-23, PKC inhibitor Go 6983 for 24 h, and the nuclear Nrf2, p-Nrf2, and Nrf2 in TCP are then measured and quantified (**K, L**) from Western blots (**J**). The protein level of nuclear Nrf2 was normalized to Histone H3; relative levels of p-Nrf2 in TCP were normalized to Nrf2; Histone H3 and GAPDH as internal controls. In **K, L** *n* = 3 biological replicates. Data are presented as the mean ± SEM. *P < 0.05, **P < 0.01, ***P < 0.001, n.s. no significant difference by one-way ANOVA with Bonferroni *post hoc* analysis.

To investigate whether NF-κB p65 suppresses Nrf2 expression by facilitating Keap1-dependent proteasomal degradation under C3a stimulation, NSCs were treated with DMSO, MG-132 (proteasome inhibitor), C3a, and C3a combined with JSH-23 (NF-κB p65 inhibitor). Whole-cell lysates were immunoprecipitated using an anti-Keap1 antibody. The co-IP results revealed that C3a significantly enhanced the co-precipitation of Nrf2 with Keap1 (Fig. 5A, B, upper blot, lane 3), correlating with a significant reduction of the total Nrf2 protein level (Fig. 5A, B, lower blot, lane 3). Notably, co-treatment with either MG-132 or JSH-23 attenuated the Keap1/Nrf2 interaction and restored Nrf2 expression (Fig. 5A, B). Furthermore, co-IP analysis further showed that C3a enhanced Nrf2 ubiquitination, an effect that was substantially suppressed by JSH-23 (Fig. 5C, D). These results suggested that C3a induces the ubiquitination-dependent degradation of Nrf2 by modulating the Keap1-Nrf2 axis *via* NF-κB p65 activation. In addition, it has been reported that NF-κB p65 can interact with Keap1 to inhibit the Nrf2-ARE pathway [24]. To explore whether NF-κB p65 directly regulates Nrf2 degradation *via* Keap1, we performed Co-IP assays using an anti-NF-κB p65 antibody. Results revealed that NF-κB p65 co-precipitated with both Keap1 and Nrf2 (Fig. S3), suggesting that NF-κB p65 may regulate Nrf2 degradation by forming a ternary complex with Keap1/Nrf2. However, the precise mechanism of interaction between NF-κB p65 and Keap1 remains to be elucidated.

In addition to ubiquitination, phosphorylation is the other significant post-translational modification of Nrf2 involved in multiple aspects of its modulation. Phosphorylation of Nrf2, a crucial signal for Nrf2 nuclear translocation, is one of the critical mechanisms of its activation and driving the Nrf2-ARE pathway at the transcriptional level. Given the pronounced effects of NF-κB p65 on both nuclear Nrf2 and p-Nrf2 protein levels (Figs 3A, 4B), we investigated whether NF-κB p65 influences Nrf2 phosphorylation. NSCs were treated with C3a alone or co-treated with JSH-23 (NF-κB p65 inhibitor), followed by detection of Nrf2-associated protein kinases *via* qRT-PCR analysis. Results showed that C3a reduced the mRNA levels of multiple protein kinases, and this was rescued by C3a+JSH-23 administration (Fig. 5E, F). Different kinases have distinct regulatory effects on Nrf2 function. For instance, PKC-mediated phosphorylation of Nrf2 at Ser40 disrupts the Keap-Nrf2 interaction and subsequent proteasomal degradation, whereas GSK-3β may negatively regulate Nrf2 activity by enhancing its nuclear export. In consideration of the negative modulation of Nrf2 by NF-κB p65, we focused on four key kinases – PKC, AKT, ERK, and AMPKα – to further explore the Nrf2 phosphorylation mediated by NF-κB p65 in this context. Firstly, to determine the optimal working concentration for each kinase activator, NSCs were treated with gradient concentrations (0, 1, 5, 10, 20, and 40 μmol/L) of the kinase activators SC-10 (PKC activator), SC79 (AKT activator), Ro67-7476 (ERK1/2 activator), and GSK621 (AMPKα activator) for 24 h. Cell viability assays revealed that all activators maintained > 85% cell viability at concentrations up to 10 μmol/L (except SC79 at ~80%), while higher doses (20–40 μmol/L) induced significantly decreased viability (< 80%) (Fig. S4A, D, G, J). Western blot analysis demonstrated dose-dependent increases in kinase phosphorylation (p-PKC, p-AKT, p-ERK1/2, and p-AMPKα) up to 10 μmol/L for all activators (Fig. S4). However, at higher concentrations (20–40 μmol/L), kinase activation either plateaued or declined, coinciding with reduced cell viability (< 80%), suggesting potential cytotoxicity at these doses (Fig. S4). Collectively, these findings indicate that 10 μmol/L is the optimal concentration for kinase activation, providing maximal phosphorylation while maintaining cell viability. Next, NSCs were separately treated with C3a alone or C3a combined with the kinase activators, and the Nrf2 activation was assessed. Compared to the Control group, treatment with C3a significantly reduced the protein levels of p-Nrf2 and nuclear Nrf2 (Fig. 5G–I). Notably, among the tested kinase activators, only co-treatment with C3a along with the PKC activator SC-10 significantly rescued the expression of p-Nrf2 and nuclear Nrf2, similar to the restorative effects found with NF-κB p65 inhibition (C3a+JSH-23) (Fig. 5G–I). Furthermore, this PKC-dependent mechanism was further confirmed by the finding that the PKC inhibitor Go 6983 abolished the restoration of p-Nrf2 and nuclear Nrf2 protein levels mediated by C3a+JSH-23 treatment (Fig. 5J–L). These results indicated that C3a/NF-κB p65 suppresses Nrf2 through two distinct but complementary pathways: (1) promoting Keap1-dependent proteasomal degradation of Nrf2; and (2) inhibiting PKC-mediated Nrf2 phosphorylation and subsequent nuclear translocation.

### Nrf2 Facilitates NSC Activation by Promoting the Clearance of UCH-L1-UPS-Mediated Protein Aggregates

**Fig. 6:**
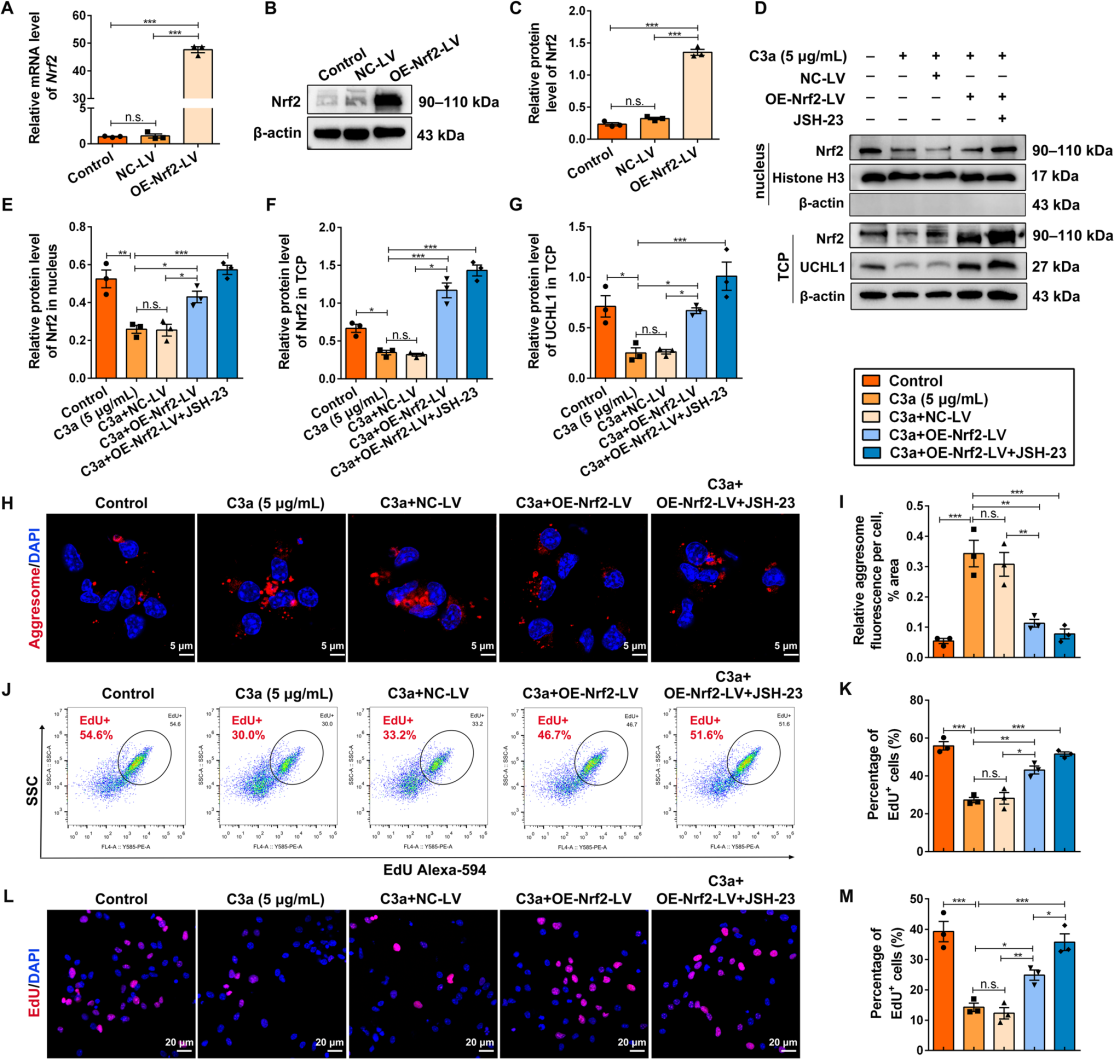
Upregulation of Nrf2 promotes NSC activation by enhancing UCHL1 expression and aggresomes clearance. **A** Relative mRNA levels of Nrf2 normalized to that of GAPDH using qRT-PCR (NSCs were treated with DMSO, negative control lentivirus (NC-LV), and Nrf2-overexpressing lentivirus (OE-Nrf2-LV). *n* = 3 biological replicates. **B, C** Western blots (**B**) and quantification (**C**) of protein levels of Nrf2 in NSCs treated with DMSO, NC-LV, and OE-Nrf2-LV. β-actin serves as the internal control. **D–G** Western blots (**D**) and quantification (**E–G**) nuclear Nrf2 protein levels normalized to Histone H3 (NSCs were administered with DMSO, C3a, or along with NC-shRNA and OE-Nrf2-LV, then the nuclear protein and TCP were extracted separately and probed by immunoblotting with anti-Nrf2, p-Nrf2, Histone H3, and β-actin; relative levels of p-Nrf2 in TCP normalized to Nrf2; relative levels of UCHL1 in TCP normalized to β-actin. Histone H3 and β-actin as internal controls. **H, I** Following treatment with DMSO, C3a, or/with NC-LV and OE-Nrf2-LV, NSCs are fixed and immune-stained for Proteostat (red) (**H**), and the Proteostat enrichment is quantified (**I**)**.** Scale bars in **H**, 5 μm. **J, K** Intracellular flow cytometry (**J**) and quantification of percentages of EdU^+^ cells (**K**) show NSC activation after treatment with DMSO, C3a, or along with NC-LV and OE-Nrf2-LV by EdU incorporation assay. **L, M** NSCs subjected to DMSO, C3a, or along with NC-LV and OE-Nrf2-LV treatments, immunostained for EdU (red) (**L**). The relative percentages of EdU^+^ cells among different groups (**M**). Scale bars in **L**, 5 μm. **A/C/E–G/I/K/M**, *n* = 3 biological replicates. Data are presented as the mean ± SEM. *P < 0.05, **P < 0.01, ***P < 0.001, n.s. no significant difference, by one-way ANOVA with Bonferroni *post hoc* analysis.

To further investigate the modulation of UCH-L1 by Nrf2, an Nrf2-overexpressing lentivirus (OE-Nrf2-LV) was constructed, and the overexpression efficiency was verified by qRT-PCR (Fig. 6A) and Western blotting analysis (Fig. 6B, C). NSCs were treated with C3a alone, C3a+NC-LV, C3a+OE-Nrf2-LV, or C3a+OE-Nrf2-LV combined with JSH-23. Compared with the C3a group, co-treatment with OE-Nrf2-LV or its combination with JSH-23 significantly increased the protein levels of nuclear Nrf2, total Nrf2, and UCH-L1, with the combined treatment exhibiting a more pronounced effect (Fig. 6D–G). Moreover, upregulation of Nrf2 by OE-Nrf2-LV or with JSH-23 resulted in less protein aggresome accumulation (Fig. 6H, I) and enhanced NSC activation (Fig. 6J–M). Consistent with this, administration of the Nrf2 activator TBHQ or along with JSH-23 both evidently promoted the nuclear Nrf2, p-Nrf2, and UCH-L1 expression after C3a treatment as well (Fig. S5A–D), with more notable effects in the treatment with TBHQ combined with JSH-23. Similarly, TBHQ alone or along with JSH-23 treatments restored the proteasome activity (Fig. S5E, F) and reduced protein aggregate accumulation (Fig. S3G, H), leading to enhanced NSC activation (Fig. S5I, J), especially in the combination treatment. Our data suggested that Nrf2 upregulation enhances NSC activation *via* the promotion of UCH-L1-UPS activity.

### UCH-L1 Is the Direct Transcriptional Target of Nrf2

**Fig. 7:**
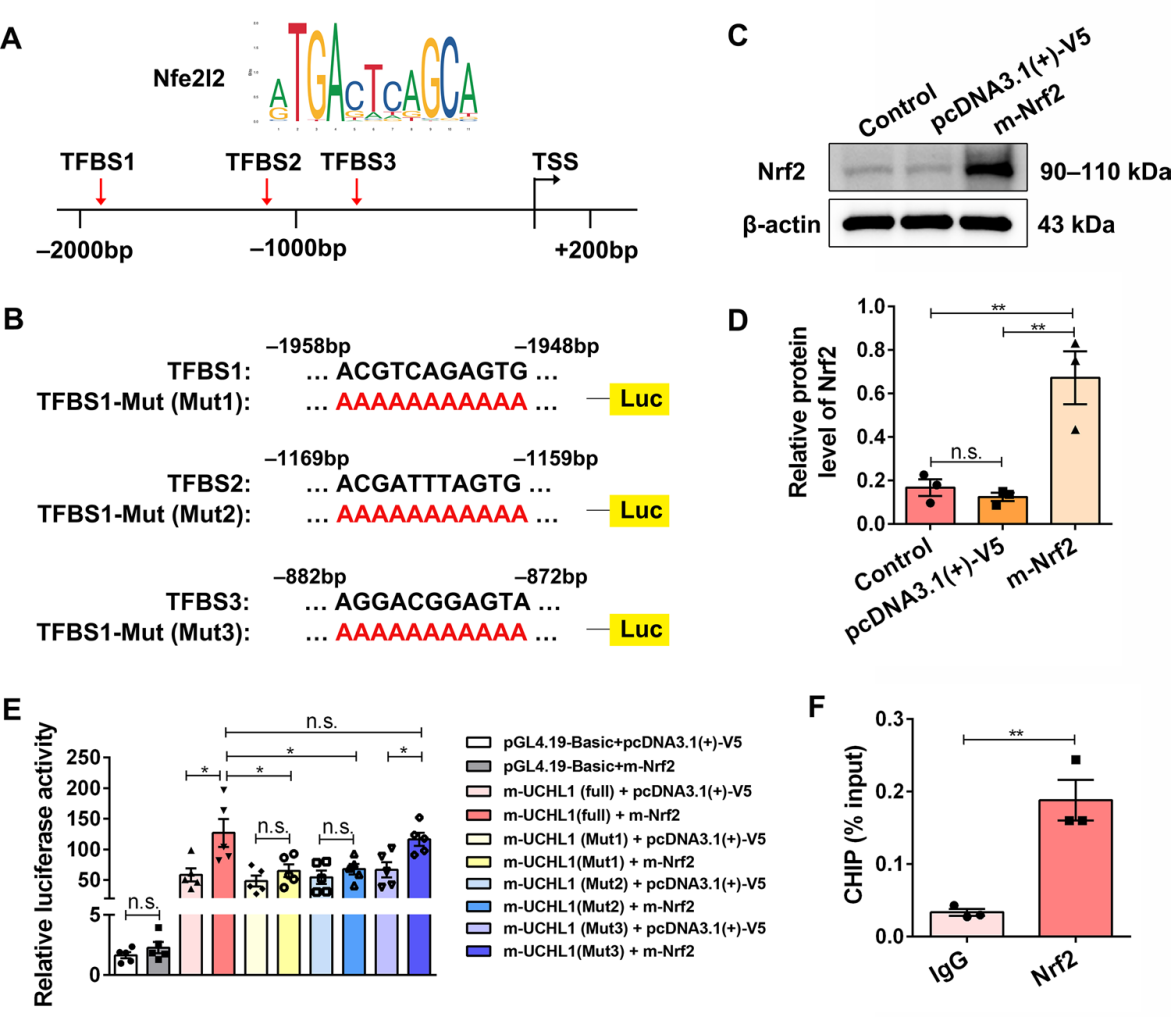
Nrf2 directly promotes the transcriptional expression of UCHL1. **A** Diagram of the Nrf2 binding motif and the putative transcription factor binding sites (TFBS) of Nrf2 on the promoter sequence of UCHL1 predicted by the JASPAR database. **B** The pGL4.19-based wild-type reporters and the corresponding mutant reporter constructs used for luciferase (Luc) assays. **C, D** Western blots (**C**) and quantification (**D**) of protein levels of Nrf2 (HEK 293T cells were transfected with DMSO, pcDNA3.1(+)-V5 plasmid (the control vector), and pcDNA3.1(+)-V5-based Nrf2 overexpression plasmid (m-Nrf2) for 48 h). β-actin serves as the internal control. *n* = 3 biological replicates. Data are presented as the mean ± SEM. **P < 0.01. n.s., no significant difference by two-tailed unpaired Student’s *t*-test. **E** Reporter activity normalized with respect to *Renilla* luciferase activity at 48 h after transfection (HEK293T cells were co-transfected with the control vector (pcDNA3.1(+)-V5), m-Nrf2, or together with the reporter constructs in the indicated combination.) *n* = 3 biological replicates. Data are presented as the mean ± SEM. *P < 0.05, n.s. no significant difference, one-way ANOVA with Bonferroni *post hoc* analysis. **F** ChIP assays of lysates from NSCs exposed to anti-Nrf2 antibody or an isotype-matched IgG. ChIP products were amplified by qRT-PCR. *n* = 3 biological replicates. Data are presented as the mean ± SEM. **P < 0.01. n.s., no significant difference, by two-tailed unpaired Student’s *t*-test.

Nrf2 is a transcription factor that participates in the antioxidant cytoprotective pathway and regulates multiple essential physiological processes to maintain cellular homeostasis. It governs the expression of numerous downstream targets such as antiapoptotic proteins, and key proteins associated with autophagy and proteasomes [32]. To explore whether Nrf2 regulates UCH-L1 expression directly, we first investigated the possibility of Nrf2 binding to the promoter sequences (−2000 to +200 bp) of UCH-L1 through JASPAR (https://jaspar.genereg.net/), a transcription factor prediction website. Results revealed three potential transcription factor binding sites of Nrf2 in the UCH-L1 promotor region (Fig. 7A). Next, we constructed a Firefly luciferase reporter plasmid with the Uch-l1 promoter sequences (−2000 to +100) (m-UCHL1(full)) and the corresponding Firefly luciferase reporter plasmids with the Uch-l1 promoter mutant sequence (m-UCHL1(Mut1/2/3)) (Fig. 7B). The plasmid m-UCHL1(full) or m-UCHL1(Mut1/2/3) was co-transfected into HEK293T cells with the Nrf2 overexpression plasmid (m-Nrf2) or its control plasmid pcDNA3.1(+)-V5, as well as the *Renilla* luciferase control plasmid. The overexpression of Nrf2 by m-Nrf2 was confirmed in HEK293T cells (Fig. 7C, D). The dual-luciferase reporter assay showed that, compared with the Control groups, the luciferase activity of m-UCHL1(full) was significantly increased by m-Nrf2 (Fig. 7E). However, this effect was abolished when m-Nrf2 was co-transfected with m-UCHL1(Mut1) or m-UCHL1(Mut2), but not m-UCHL1(Mut3) (Fig. 7E), indicating that Nrf2 might modulate the transcriptional expression of UCHL1 directly through binding to TFBS1 and TFBS2. ChIP analysis further verified the combination of Nrf2 with the promoter region of UCH-L1 (Fig. 7F). Taken together, these data suggested that Nrf2 directly promotes the transcriptional expression of UCH-L1, thus enhancing proteasome activity and NSC activation.

### C3a Inhibits Spinal Cord NSC Activation *via* the NF-κB p65/Nrf2/UCH-L1 Pathway following SCI

**Fig. 8:**
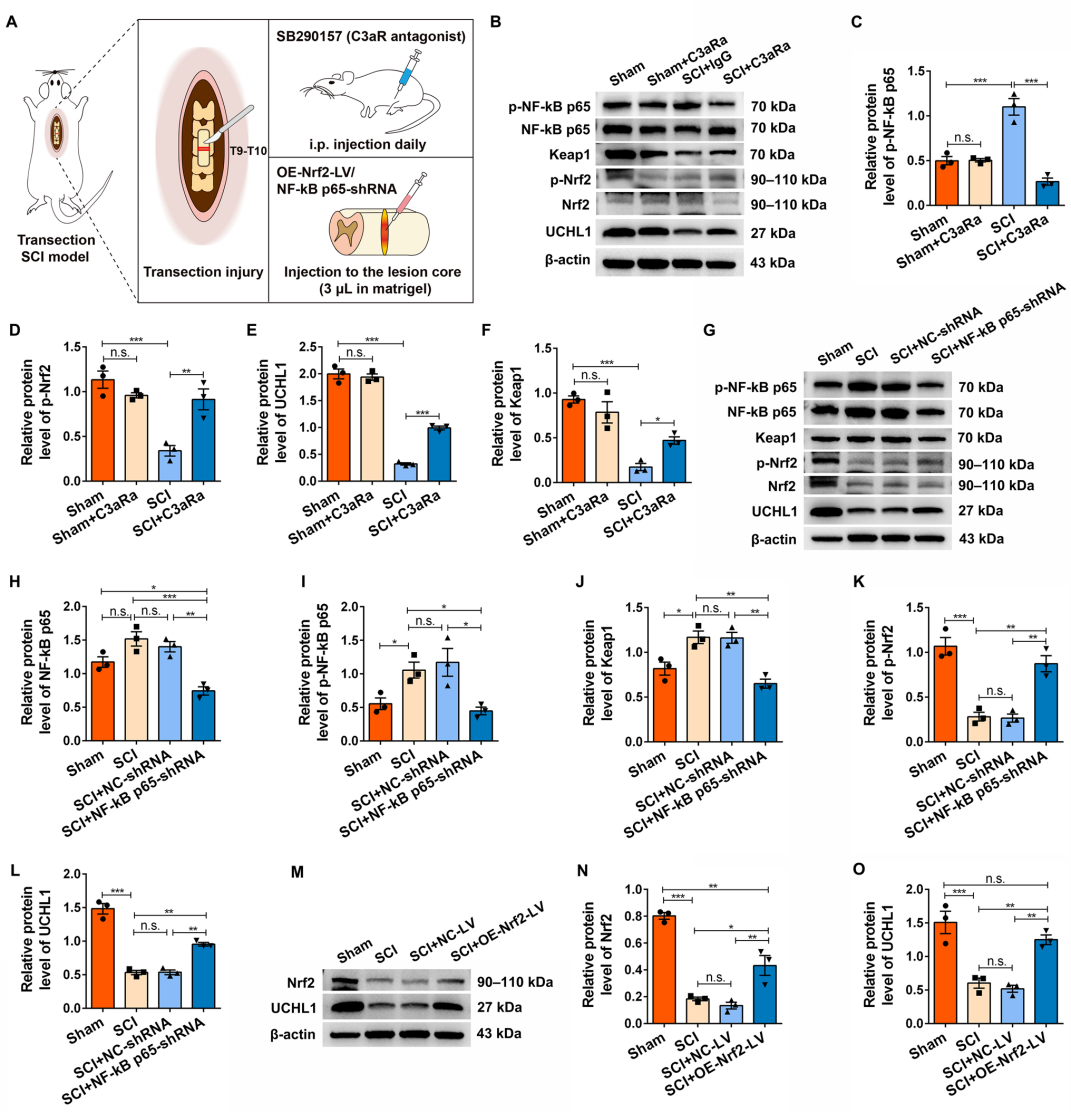
C3a may inhibit spinal cord NSC activation after SCI through the NF-κB p65/Nrf2/UCHL1 pathway.** A** Diagram of the construction of a complete transection SCI model, and the administration of C3aR antagonist or lentivirus after SCI. **B–F** The NF-κB p65/Keap1/Nrf2/UCHL1 pathway in lesioned tissues was quantified using Western blot analysis at 7 days after treatment with the C3aR antagonist post-SCI. Relative expression of p-NF-κB p65 and p-Nrf2 normalized to NF-κB p65 and Nrf2 separately. β-actin as the internal control. **G–L** SCI mice treated with NF-κB p65-shRNA were sacrificed at seven days post-injury, and the Protein levels in the NF-κB p65/Keap1/Nrf2/UCHL1 pathway were quantified by Western blots. Relative expression of p-NF-κB p65 and p-Nrf2 normalized to NF-κB p65 and Nrf2 separately. β-actin as the internal control. **M–O** Seven days post-surgery, SCI mice injected with OE-Nrf2-LV are sacrificed, and the protein levels of Nrf2 and UCHL1 are quantified by Western blots. β-actin as the internal control. In **C–F, H–L, N, O**, *n* = 3 independent animals. Data are presented as the mean ± SEM. *P < 0.05, **P < 0.01, ***P < 0.001, n.s. no significant difference, by one-way ANOVA with Bonferroni *post hoc* analysis.

**Fig. 9:**
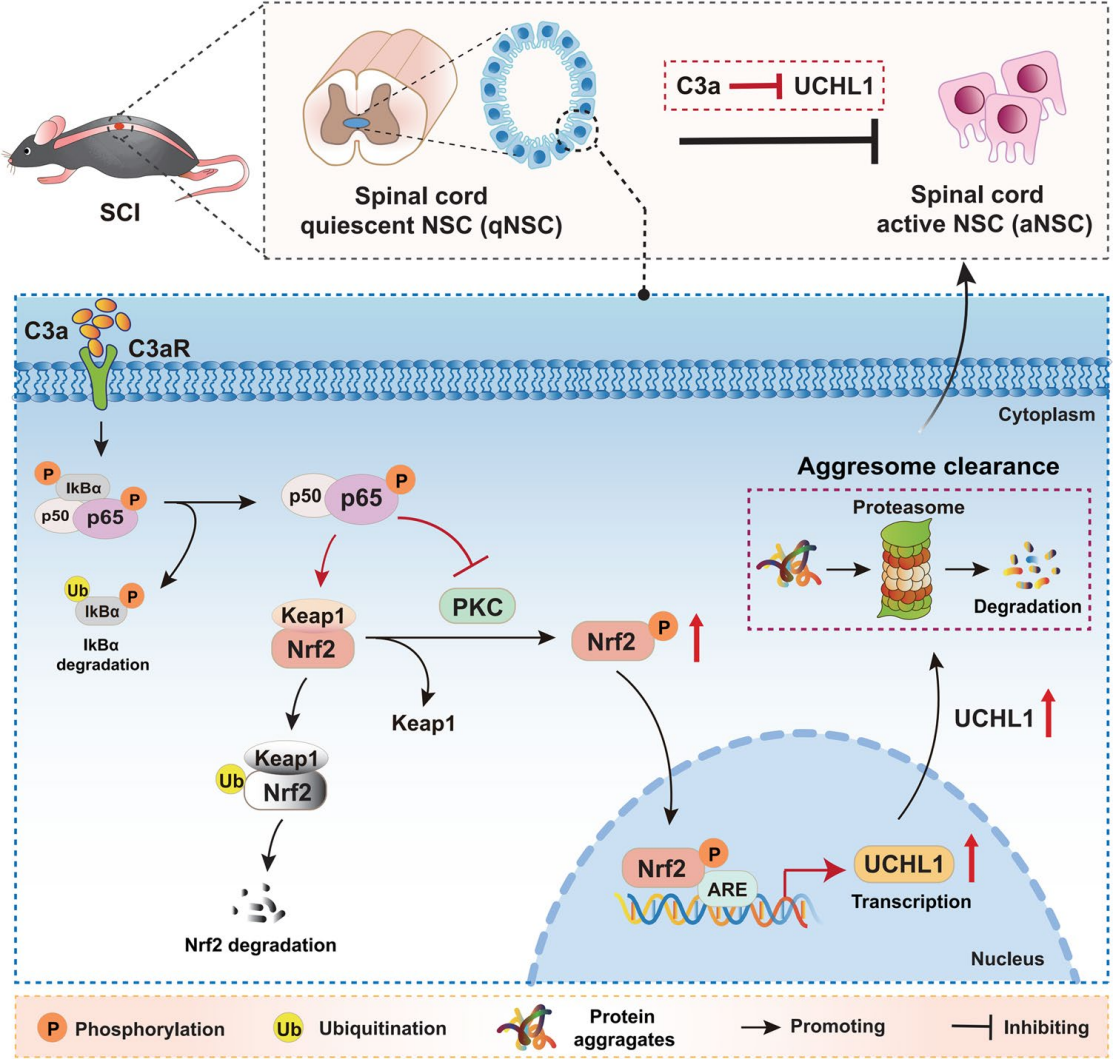
Complement C3a suppresses spinal cord NSC activation post-SCI by inhibiting UCHL1 through the NF-κB p65/Nrf2 signaling pathway. C3a/C3aR signaling inhibits spinal NSC activation after SCI through NF-κB p65-mediated suppression of the Nrf2/UCHL1 pathway. Mechanistically, NF-κB p65 inhibits Nrf2 through a dual mechanism: (1) enhancing Keap1-dependent ubiquitination degradation of Nrf2; (2) suppressing PKC-mediated Nrf2 phosphorylation and nuclear translocation. Moreover, UCHL1 is the direct transcriptional target of Nrf2 during NSC activation. Either blockade of C3a/C3aR signaling, NF-κB p65 Knockdown, or Nrf2 overexpression rescues UCHL1 expression and impairs proteasome activity inhibited by C3a, contributing to protein aggregate clearance and spinal cord NSC activation.

Our previous study has demonstrated that both upregulation of UCH-L1 and blockade of C3a/C3aR signaling significantly promote spinal cord NSC activation by restoring the UCH-L1 level post-SCI [10], but the molecular mechanism by which C3a modulates UCH-L1 remains elusive. The current *in vitro* findings suggest that C3a suppresses NSC activation by UCH-L1 downregulation through the NF-κB p65/Nrf2 pathway. To validate these mechanisms *in vivo*, we established a T10 transection SCI model and implemented three intervention strategies (Fig. 8A): (1) Daily i.p. injection of the C3aR antagonist SB290157 (C3aRa) to block C3a/C3aR signaling; (2) Local injection of NF-κB p65-shRNA lentivirus to knock down NF-κB p65; (3) Local injection of OE-Nrf2-LV to upregulate Nrf2. At 7 days post-SCI, we found significant upregulation of p-NF-κB p65 and Keap1 protein levels, accompanied by corresponding decreases in p-Nrf2 and UCH-L1 expression (Fig. 8B–F). Notably, C3aR antagonism reversed these effects, increasing p-Nrf2 and UCH-L1 levels while suppressing the NF-κB p65/Keap1 pathway (Fig. 8B–F). Consistent with these findings, NF-κB p65 knockdown similarly elevated p-Nrf2 and UCH-L1 expression while reducing Keap1 levels (Fig. 8G–L). Furthermore, Nrf2 overexpression significantly enhanced UCHL1 expression (Fig. 8M–O). Building upon our previous evidence that UCH-L1 is a critical regulator of NSC activation, these *in vivo* results corroborate our *in vitro* data, supporting the conclusion that C3a inhibits spinal cord NSC activation post-SCI through NF-κB p65-mediated suppression of the Nrf2/UCH-L1 pathway (Fig. 9).

## Discussion

Mobilization of the quiescent endogenous NSCs to activate and generate newborn neurons holds a promising strategy for SCI repair. In our previous studies, we have found that the complement C3a suppresses NSC proliferation post-SCI by inhibiting the key deubiquitinating enzyme UCH-L1-UPS-mediated protein aggregate clearance. In the present study, we further reveal the molecular mechanisms concerning C3a-mediated inhibition of spinal cord NSC activation through this pathway, which advances our understanding of the potential effects of the complement system on NSC biology. Using lentiviral vector tools and small molecule drugs, we revealed that C3a/C3aR signaling suppressed UCH-L1-related NSC activation *via* the NF-κB p65/Nrf2 pathway. Both NF-κB p65 downregulation and Nrf2 upregulation enhanced the ability of NSCs to activate by promoting UCH-L1 expression and proteasome activity. Mechanistically, C3a-activated NF-κB p65 inhibits Nrf2 by a dual mechanism: promoting the Keap1-independent ubiquitination degradation of Nrf2 and blocking PKC-mediated Nrf2 phosphorylation and nuclear translocation. Notably, we identified UCH-L1 as a direct transcriptional target of Nrf2, revealing a novel mechanism by which Nrf2 promotes NSC activation. These findings collectively suggested that C3a inhibits UCH-L1-proteasome-mediated spinal cord NSC activation through the NF-κB p65/Nrf2 pathway.

The complement cascade, a highly conserved signaling pathway crucial for innate immunity, plays essential roles in neuroinflammation and CNS disorders. Accumulating evidence has demonstrated the activation of the C3a/C3aR signaling pathway in various CNS injuries and neurodegenerative diseases [13, 14]. The aberrant activated microglia aggravate white matter injury and cognitive deficits *via* the C3-C3aR pathway during chronic brain hypoperfusion [33]. C3a-C3aR interactions also mediate inflammatory cell infiltration, cytokine secretion, myelin clearance, and neuronal death after SCI, leading to the progression of many secondary injury-associated pathways [34]. C3 deficiency promotes neurite outgrowth, neuronal viability, and axon regeneration post-SCI [16, 35]. Consistent with this, in our previous study [10], the increased expression of C3a, which may be secreted from reactive astrocytes, was also found post-SCI. C3 is also closely involved in NSC fate determination and the subsequent neurogenesis. Researchers have reported that C3a regulates enteric neural crest cell migration in a N-cadherin-dependent process [36] and that it modulates neurogenesis by directly affecting the migration and neuronal differentiation of NPCs *in vitro* [37]. We have previously shown that C3a inhibits the proliferation and neuronal differentiation of NSCs derived from fetal brain by suppressing UCHL-UPS-associated protein aggresome clearance *in vitro* [10]. In line with this, *via* evaluating the quiescent NSC activation response to growth factors, we found here that C3a suppressed the ability of spinal cord NSCs to activate through the proteasome-mediated aggresome as well.

Increasing evidence highlights the pleiotropic functions of C3 in orchestrating diverse immune and inflammatory pathways. As a critical modulator of inflammation, NF-κB p65 undergoes phosphorylation upon activation. Notably, intracellular C3 has been shown to activate NF-κB p65 to trigger cell-autonomous immunity during infection [18]. C3a/C3aR signaling markedly promotes T-cell proliferation and IL-17α expression mediated by NF-κB p65 activation [38]. The complement C3a‑C3aR and C5a‑C5aR pathways both enhance the viability and inflammation of human retinal pigment epithelium cells by targeting the NF‑κB signaling pathway [19]. Consistent with this, in the present study, C3a activated NF-κB p65 *via* binding to C3aRs during NSC activation; moreover, genetic knockdown of NF-κB p65 or pharmacological inhibition (JSH-23, an NF-κB p65 inhibitor) both partially restored the impaired proteasome activity and enhanced NSC activation by UCH-L1 upregulation. Our results suggest that C3a modulates NSC activation through NF-κB p65-dependent regulation of UCH-L1, expanding our understanding of complement signaling in neural regeneration.

The hostile post-injury microenvironment, characterized by chronic inflammation and oxidative stress, critically compromises NSC survival and fate determination. NF-κB p65 and Nrf2, the two important transcription factors separately responsible for inflammatory responses and oxidative stress, exhibit functional antagonism while maintaining intricate crosstalk in multiple cellular processes, including cell death, inflammatory injury, and neurotoxicity [31, 39]. Substantial evidence shows that NF-κB p65 modulates the expression of target genes downstream from Nrf2 by regulating its transcriptional expression and activity. More importantly, as a key modulator of ROS levels and mitochondrial function, Nrf2 has profound effects on NSC self-renewal and fate determination. Deficiency of Nrf2 impairs the proliferation and neurogenesis of NPCs in the hippocampus [26]. Nrf2-dependent retrograde signaling is indispensable for the NSC fate determination mediated by mitochondrial dynamics [27]. Here, we found that C3a-mediated NF-κB p65, evidenced by the increased nuclear NF-κB p65 translocation and p-NF-κB p65 levels, significantly inhibited the expression of Nrf2 and UCH-L1, leading to protein aggregate accumulation and remarkable suppression of NSC activation *in vitro*. Notably, using the SCI mouse model, we further confirmed that blockade of C3a/C3aR signaling, NF-κB p65 knockdown, and Nrf2 overexpression *in vivo* all significantly enhanced UCH-L1 expression post-SCI. Together, these findings indicate that C3a suppresses spinal cord NSC activation post-SCI through NF-κB p65-mediated inhibition of the Nrf2/UCH-L1 axis.

Current understanding identifies two predominant regulatory mechanisms controlling Nrf2 activity: (1) Keap1-dependent ubiquitin-proteasomal degradation and (2) phosphorylation-mediated nuclear translocation. The former regulates cytoplasmic Nrf2 stability, while the latter modulates Nrf2-ARE binding and nuclear export [40]. It has been documented that NF-κB p65 could interact with Keap1 [24] or selectively remove CBP from Nrf2 to antagonize the Nrf2-ARE pathway [25]. In this study, we found that the activated NF-κB p65 induced by C3a promoted Keap1 expression, subsequently enhanced the interaction between Keap1 with Nrf2 and Nrf2 ubiquitination, resulting in the remarkable reduction of Nrf2. Moreover, a ternary complex of NF-κB p65 with Keap1/Nrf2 was detected using Co-IP analysis. These findings indicate that NF-κB p65 reduces Nrf2 activity by affecting Keap1-dependent Nrf2 proteasomal degradation, but the precise binding mechanism between NF-κB p65 and Keap1 remains to be further investigated. In addition, both the expression of p-Nrf2 and nuclear Nrf2 were also decreased following C3a treatment, which was inversely rescued by a C3aR antagonist, NF-κB p65 knockdown, or JSH-23, implying that the C3a/NF-κB p65 pathway regulates Nrf2 phosphorylation. PKC, one of the typical protein kinases that modifies Nrf2, functions to phosphorylate Nrf2 and release it from Keap1. Mounting evidence has demonstrated that PKC directly phosphorylates Ser40 within the Neh2 domain of Nrf2 to cause the dissociation of Nrf2 from its repressor Keap1, thus enhancing its nuclear localization and transcription activity [41, 42]. However, it has also been reported that PKC prevents the Keap1-mediated proteasomal degradation of Nrf2 but has no effect on Nrf2 nuclear translocation and its binding to the ARE sequence [43]. In the present study, the PKC activator SC-10 restored the decrease of p-Nrf2 and nuclear Nrf2 induced by C3a; in return, the PKC inhibitor Go 6983 counteracted the rescue effects following combination with C3a and JSH-23 treatment, indicating that NF-κB p65 inhibits Nrf2 activation by suppressing PKC-mediated Nrf2 phosphorylation in this context. Taken together, these data suggest that NF-κB p65 inhibits Nrf2 activity through a dual mechanism that promotes the Keap1-dependent proteasomal degradation of Nrf2 and inhibits its phosphorylation activation mediated by PKC. In spite of the evaluation of nuclear NF-κB p65 levels here, one of the signs of NF-κB p65 activation, the transcriptional regulation response mediated by NF-κB p65 activation, was not further evaluated. Therefore, the precise transcriptional mechanisms by which NF-κB p65 regulates Nrf2 through these dual pathways remain unclear. In addition, we cannot entirely rule out the possibility that NF-κB p65 may also inhibit Nrf2 activity by competitively depriving the CREB binding protein of Nrf2.

As a master regulator of cellular defense mechanisms, Nrf2 orchestrates the expression of numerous cytoprotective genes. Beyond its canonical role in antioxidant stress responses, Nrf2 is also involved in diverse physiological processes, including autophagy, intermediary metabolism, stem cell quiescence, the unfolded protein response, and other cellular processes [32]. Particularly relevant to protein homeostasis, Nrf2 enhances proteasome functions by upregulating multiple proteasome subunits, leading to the clearance of abnormal proteins that otherwise interfere with cellular functions [29, 30]. In keeping with these studies, we found that Nrf2 upregulation promoted UCH-L1 expression and proteasome activity, and enhanced the removal of protein aggregates correlated with improved NSC activation. Given the crucial roles of the UCHL1-UPS axis in NSC activation, as we have previously reported, the target regulation of UCH-L1 by Nrf2 was further investigated. Notably, we found that Nrf2 can directly promote the transcriptional expression of UCH-L1 by binding to its promoter region *via* dual-luciferase reporter assay and CHIP analysis. In this study, we identified UCH-L1 as a novel transcriptional target of Nrf2, highlighting the significance of Nrf2/UCHL1 signaling in promoting NSC activation.

In summary, this study elucidates a novel molecular mechanism by which the complement C3a inhibits spinal cord NSC activation through the UCHL1-UPS pathway. C3a activated NF-κB p65, which inhibits Nrf2/UCHL1 signaling, resulting in decreased proteasome activity and accumulation of protein aggresomes, consequently impairing the ability of NSCs to activate. Mechanistically, NF-κB p65 suppressed Nrf2 activity *via* a dual mechanism: promoting the Keap1-dependent ubiquitination degradation of Nrf2, and inhibiting its phosphorylation and nuclear translocation. Furthermore, Nrf2 directly promoted the transcriptional expression of UCHL1, therefore contributing to the enhanced NSC activation by facilitating protein aggregate clearance through the UPS. These findings highlight the critical roles of the NF-κB p65/Nrf2/UCHL1 pathway in spinal cord NSC activation, providing new mechanistic insights into complement-mediated NSC inhibition and potential therapeutic targets for mobilizing endogenous NSC activation for SCI repair. Further investigations are needed to elucidate how protein aggregates mechanistically affect NSC activation and fate determination.

## Supplementary Information

Below is the link to the electronic supplementary material.

Supplementary file1 (PDF 1206 KB)
