# Supplementary material for: Complement C3a Suppresses Spinal Cord Neural Stem Cell Activation by Inhibiting UCHL1 via the NF-κB p65/Nrf2 Pathway
Source: Neurosci Bull. 2025 Oct 3;42(1):153–74. doi: 10.1007/s12264-025-01488-z (PMC12789345; doi:10.1007/s12264-025-01488-z)
Supplement: Supplementary file 1 — Supplementary file1 (PDF 1206 KB) [file 12264_2025_1488_MOESM1_ESM.pdf]

# Supplementary Information

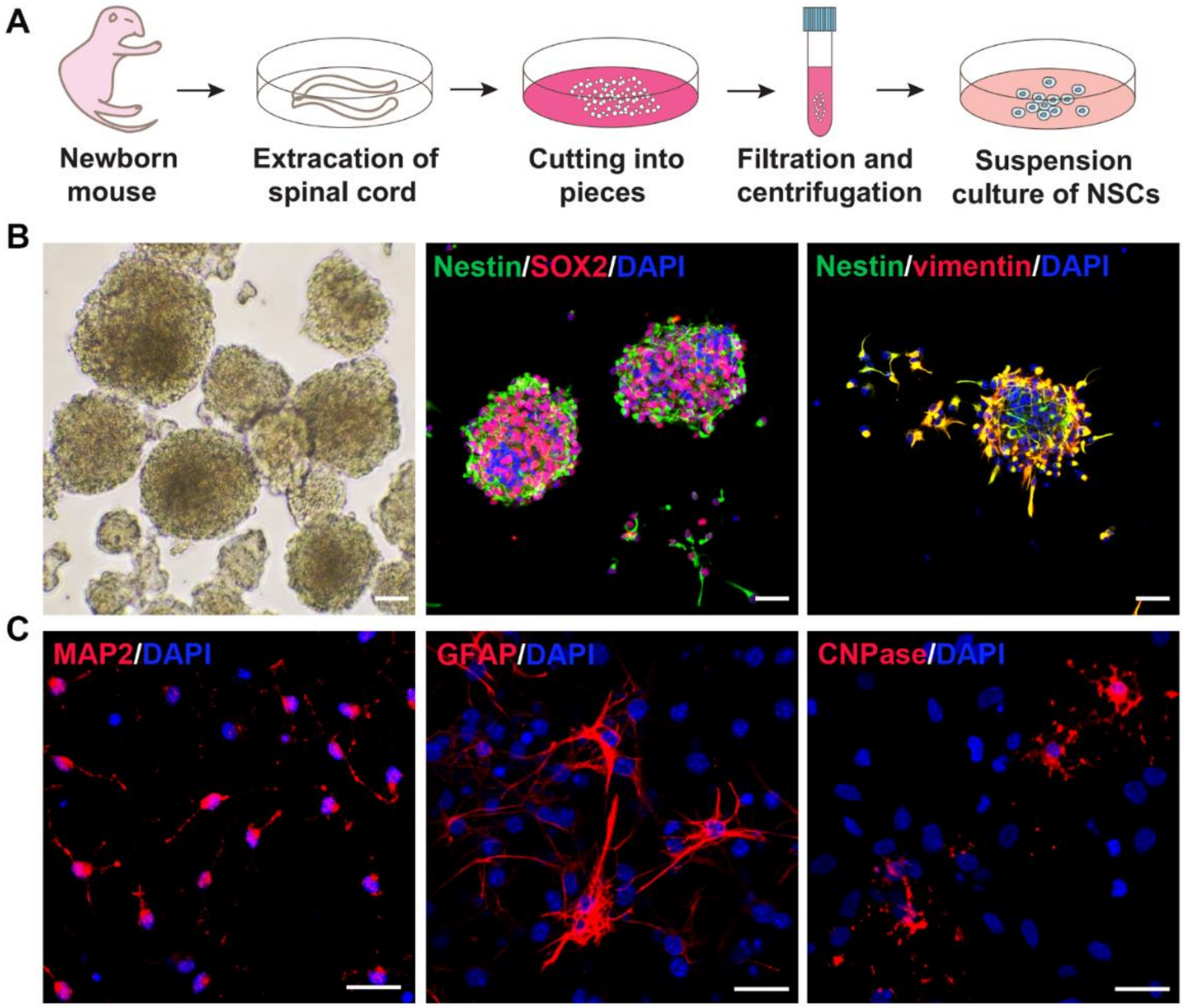


**Fig.S1** Extraction and Identification of Spinal Cord NSCs. **A** An Illustration showing the isolation and extraction of spinal cord NSCs derived from the postnatal mouse. **B** Spinal cord NSCs exhibited robust self-renewal and proliferation capacity in vitro. NSCs formed the representative neurospheres in vitro and were positive for the typical NSC markers, including Nestin, SOX2, and vimentin. Scale bars, 20 μm. **C** Spinal cord NSCs showed multiple-directional differentiation ability in vitro, which differentiated into the typical cell types, including neurons ($MAP2^+$), astrocytes ($GFAP^+$), and oligodendrocytes ($CNPase^+$). Scale bar, 20 μm.

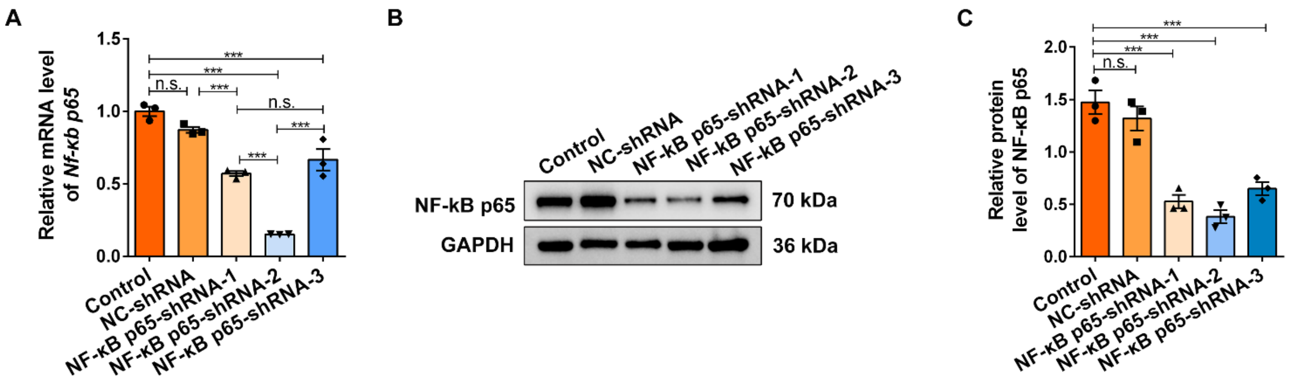


**Fig.S2** The Knockdown of NF-kB p65 by Lentivirus. **A–C** Three different knockdown-lentivirus encoded NF-kB p65 were constructed and used to infect NSCs for 48 h. The mRNA and protein levels of NF-kB p65 were then confirmed using qRT-PCR (**A**) and Western blot analysis (**B–C**), separately. NF-kB p65 shRNA-2 showed the best knock-down efficiency and was selected for the following experiments. GAPDH serves as the internal control. (**A, C**) $n = 3$ different biological replicates. Each data is presented as Mean ± SEM. $P$-values (***$P < 0.001$, n.s. not significant) are determined by one-way ANOVA with Bonferroni post hoc analysis.

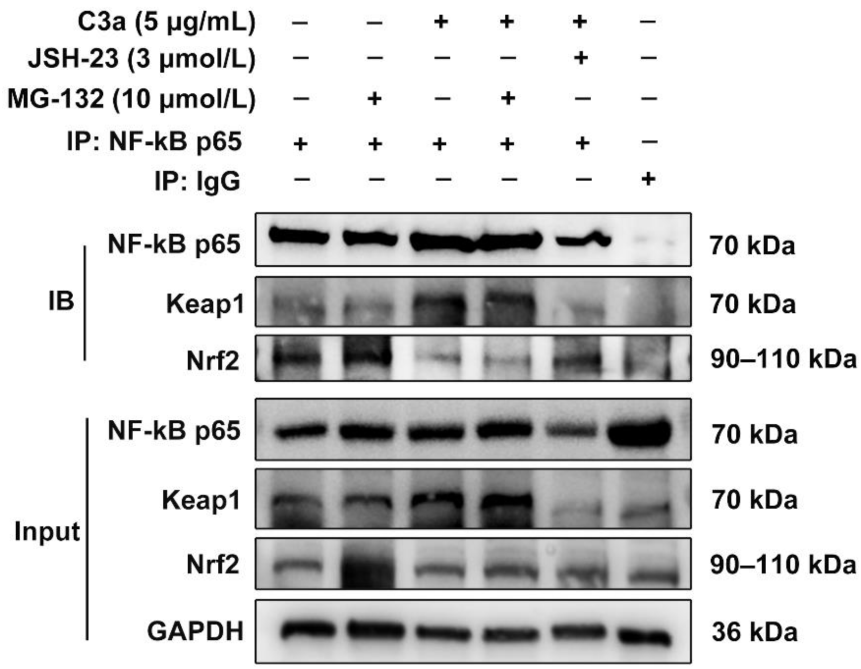


**Fig.S3** Co-IP analysis showed a ternary complex formation of NF-κB p65/Keap1/Nrf2. NSCs were pretreated with MG-132 (10 μmol/L) for 4 h, before 24 h treatment with DMSO, C3a, or/with NF-κB p65 inhibitor JSH-23 in the continued presence or absence of MG132. The whole NSCs lysates were

subjected to immunoprecipitation with anti-NF-κB p65 and analyzed by immunoblotting using antibodies against NF-κB p65, Keap1, and Nrf2. Input: 10% of the cell lysate used for immunoprecipitation. IgG as a negative control. GAPDH served as an internal control.

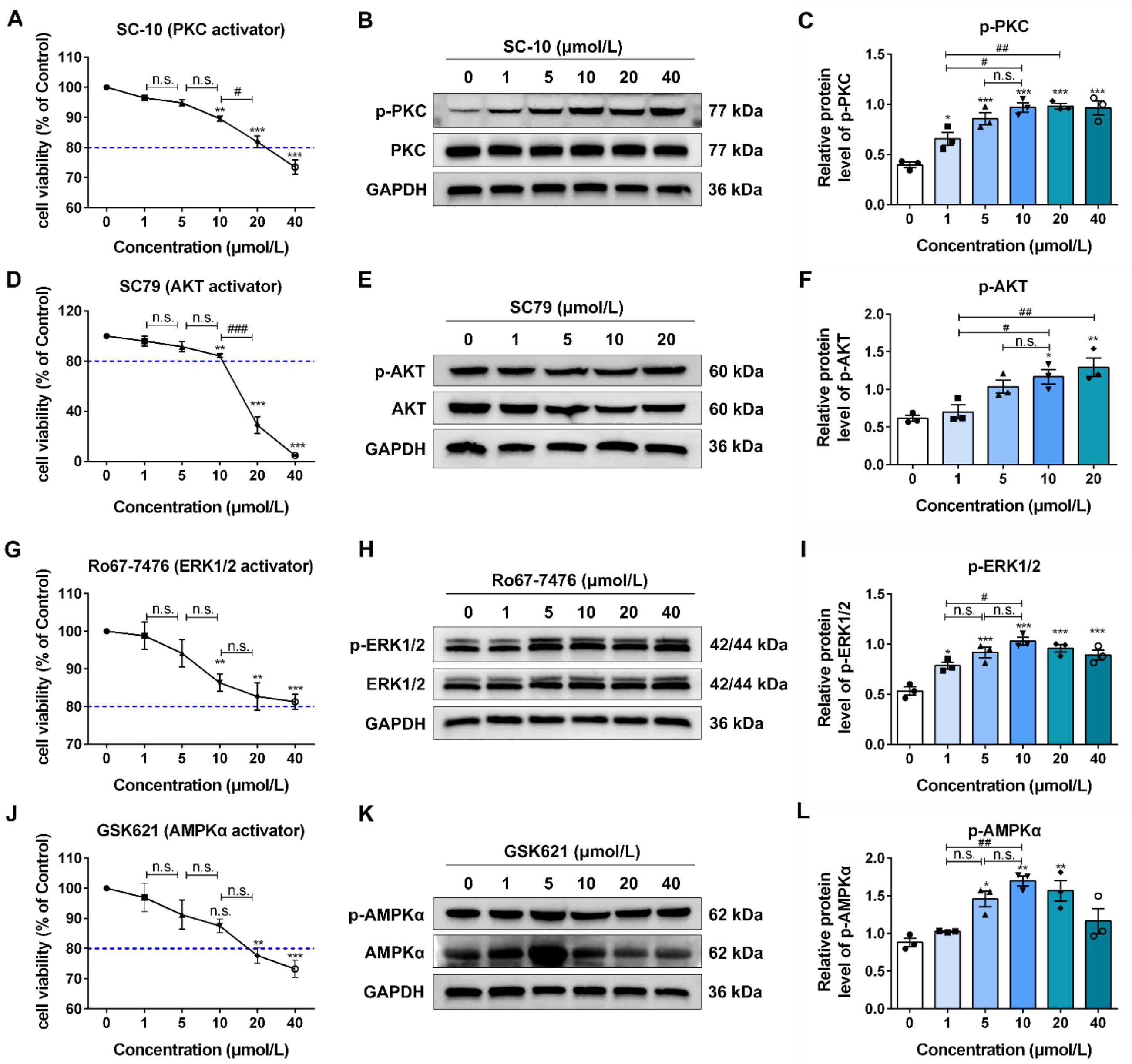


**Fig.S4** Determination of optimal protein kinase activator concentrations. **A** NSCs were treated with SC-10 (PKC activator) at gradients of 0, 1, 5, 10, 20, and 40 μmol/L for 24 h, and the cell viability was tested using the CCK8 assay. **B–C** The protein levels of p-PKC, PKC, and GAPDH were measured and quantified using Western blot analysis. Relative expressions of p-PKC normalized to PKC. GAPDH as the internal control. **D–F** Cells were administered with **SC79** (AKT activator) at different

concentrations for 24 h. The cell viability was tested using CCK8 assay (**D**), and the protein levels of p-AKT, AKT, and GAPDH were measured and quantified by Western blotting (**E−F**). Relative expressions of p-AKT normalized to AKT. GAPDH as the internal control. **G–I** Cells were incubated with Ro67-7476 (ERK1/2 activator) for 24h, then the cell viability was tested using CCK8 assay (**G**), and the protein levels of p-ERK1/2, ERK1/2, and GAPDH were measured and quantified by Western blotting (**H–I**). Relative expressions of p-ERK1/2 normalized to ERK1/2. GAPDH as the internal control. **J–L** After treatment with GSK621 (AMPKα activator) for 24h, the cell viability was evaluated by CCK8 assay (**G**), and the protein levels of p-AMPKα, AMPKα, and GAPDH were measured and quantified via Western blotting (**H–I**). Relative expressions of p-AMPKα normalized to AMPKα. GAPDH as the internal control. (**A/C/D/F/G/I/J/L**) $n = 3$ different biological replicates. Each data is presented as Mean ± SEM. For all panels, *P*-values (*$P < 0.05$, **$P < 0.01$, ***$P < 0.001$, n.s. not significant) are determined by one-way ANOVA with Bonferroni post hoc analysis.

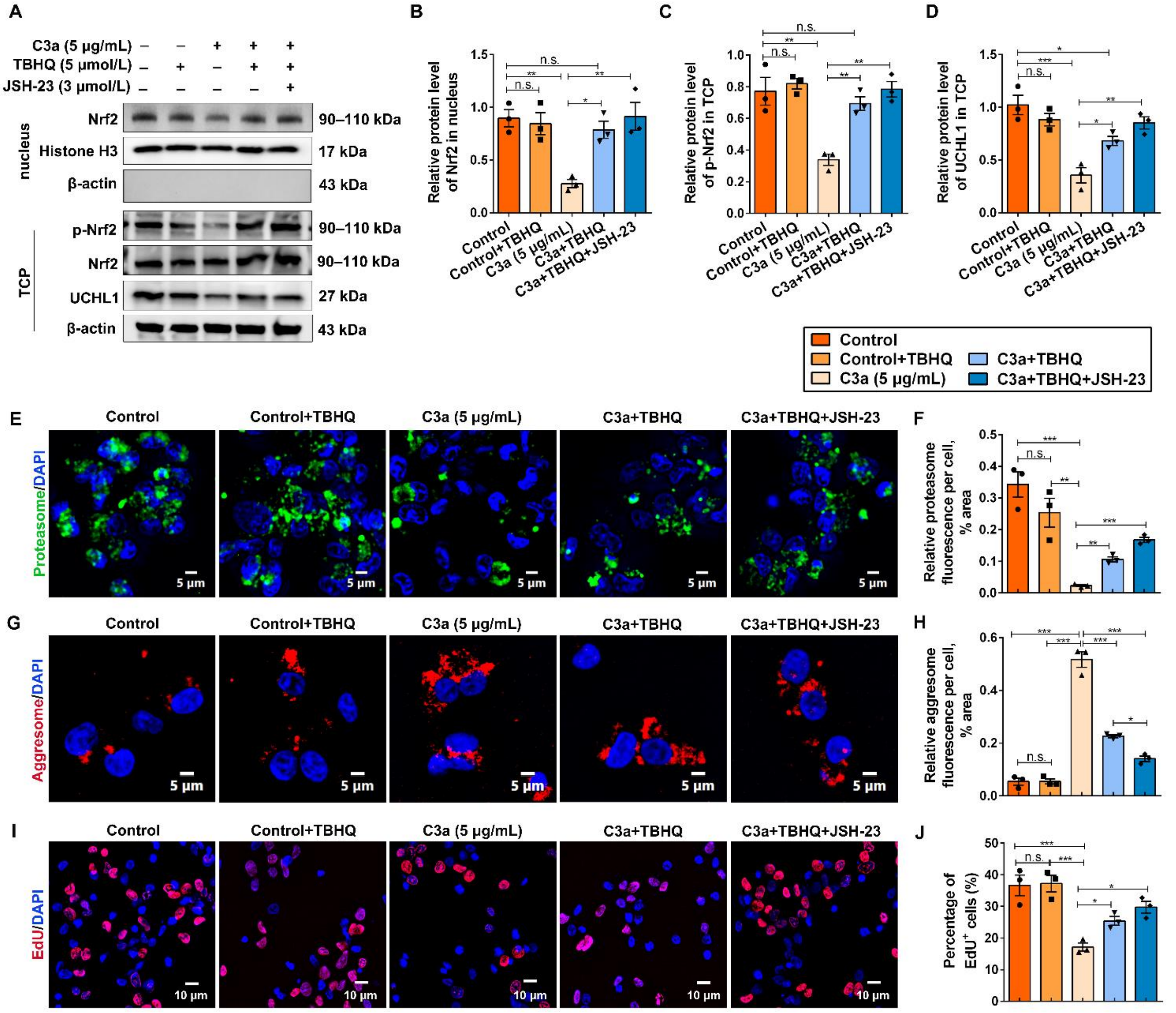


**Fig.S5** Nrf2 Activator, TBHQ, Facilitated NSC Activation by Enhancing Protein Aggregates Removal Modulated by UCHL1-UPS. **A** NSCs were treated with DMSO, C3a, or/with Nrf2 activator TBHQ and NF-κB p65 inhibitor JSH-23 for 24 h, then the nuclear proteins and TCP in NSCs were extracted and probed by western blotting for specific antibodies. **B–D** The semiquantitative results of the blottings in A. The protein level of nuclear Nrf2 normalized to Histone H3. Relative levels of p-NRF2 in TCP normalized to Nrf2. Relative levels of UCHL1 in TCP normalized to β-actin. Histone H3 and β-actin serve as the internal controls. *n* = 3 different biological replicates. **E–F** Representative immunofluorescence images and quantification (**F**) of proteasome activity in NSCs stained with proteasome probes (proteasome, green) after treatment with DMSO, C3a, or/with TBHQ and JSH-23 for 24 h. Scale bars (**E**), 5 μm. (**F**) *n* = 3 different biological replicates. **G–H** Following treatment with

DMSO, C3a, or/with Nrf2 TBHQ and JSH-23 for 24 h, NSCs were fixed and stained for aggregated proteins with the dye Proteostat (red). Quantification is shown in H. Scale bars (**G**), 5 µm. (**H**) $n$ = 3 different biological replicates. **I–J** NSCs were treated with DMSO, C3a, or/with TBHQ and JSH-23 for 24 h. During the treatment, cells were incubated with EdU overnight, then immunostained and analyzed (**J**) for the percentage of $EdU^+$ NSCs. Scale bars (**I**), 10 µm. (**J**) $n$ = 3 different biological replicates. Each data is presented as Mean ± SEM. For all panels, $P$-values (*$P < 0.05$, **$P < 0.01$, ***$P < 0.001$, n.s. not significant) are determined by one-way ANOVA with Bonferroni post hoc analysis.

**Table S1.** Materials and antibodies used in this study.

| REAGENT OR RESOURCE | SOURCE | IDENTIFIER |
|---|---|---|
| Antibodies | | |
| Mouse monoclonal anti-Nestin (Rat-401) | Cell Signaling Technology | Cat# 4760, RRID: AB_2235913 |
| Rabbit monoclonal anti-GFAP (E4L7M) | Cell Signaling Technology | Cat# 80788, RRID: AB_2799963 |
| Rabbit monoclonal anti-UCHL1 (D3T2E) | Cell Signaling Technology | Cat# 13179, RRID: AB_2798141 |
| Rabbit monoclonal anti-CNPase (D83E10) | Cell Signaling Technology | Cat# 5664, RRID: AB_10705455 |
| Chicken polyclonal anti-MAP2 | Abcam | Cat# ab5392, RRID: AB_2138153 |
| Rabbit polyclonal anti-SOX2 | Abcam | Cat# ab97959, RRID: AB_2341193 |
| Rabbit monoclonal anti-Vimentin (EPR3776) | Abcam | Cat# ab92547, RRID: AB_10562134 |
| Mouse monoclonal anti-C3aR | Santa Cruz | Cat# sc-133172, RRID: AB_2066736 |
| Rabbit monoclonal anti-Ubiquitin (EPR8830) | Abcam | Cat# ab134953, RRID: AB_2801561 |

| | | |
|---|---|---|
| Mouse monoclonal anti-NF-κB p65 (L8F6) | Cell Signaling Technology | Cat# 6956, RRID: AB_784821 |
| Rabbit polyclonal anti-NRF2 (D1Z9C) | Cell Signaling Technology | Cat# 12721, RRID: AB_443209 |
| Rabbit polyclonal anti-NF-κB p65 | Servicebio | Cat# GB11997 |
| Rabbit polyclonal anti-Phospho-NF-kB p65 | Servicebio | Cat# GB113882 |
| Rabbit polyclonal anti-NRF2 | Servicebio | Cat# GB115673 |
| Rabbit polyclonal anti-NRF2 (phospho S40) | Abcam | Cat# ab76026, RRID: AB_1524049 |
| Rabbit polyclonal anti-Keap1 | Servicebio | Cat# GB113747 |
| Rabbit polyclonal anti-Keap1 | Proteintech | Cat# 10503-2-AP |
| Rabbit polyclonal anti-PKC | Abcam | Cat# ab32376 |
| Rabbit polyclonal anti-Phospho-PKC | Abcam | Cat# ab23513 |
| Rabbit polyclonal anti-AKT | Cell Signaling Technology | Cat# 9272 |
| Rabbit polyclonal anti-Phospho-AKT | Cell Signaling Technology | Cat# 4060 |
| Rabbit polyclonal anti-ERK1/2 | Cell Signaling Technology | Cat# 4695 |
| Rabbit polyclonal anti-Phospho- ERK1/2 | Cell Signaling Technology | Cat# 4370 |
| Rabbit polyclonal anti-MAPKα | Cell Signaling Technology | Cat# 2532 |
| Rabbit polyclonal anti-Phospho- MAPKα | Cell Signaling Technology | Cat# 2535 |
| Rabbit monoclonal anti-GAPDH | Cell Signaling Technology | Cat# 5174, RRID: AB_10622025 |
| Mouse monoclonal anti-β-Actin | Cell Signaling Technology | Cat# 58169, RRID: AB_2750839 |
| Rabbit polyclonal anti-Histone H3 | Proteintech | Cat# 39155, RRID: AB_2561020 |

| | | |
|---|---|---|
| DAPI | Sigma-Aldrich | Cat# D9542 |
| Goat anti-chicken IgG (H&L) Secondary Antibody, Alexa Flour 647 conjugate | Abcam | Cat# ab150171 |
| Goat anti-mouse IgG (H+L) Cross-Adsorbed Secondary Antibody-Alexa Flour 488 conjugate | Thermo Fisher Scientific | Cat# A-11001, RRID: AB_2534069 |
| Goat anti-rabbit IgG (H+L) Cross-Adsorbed Secondary Antibody, Alexa Flour 647 conjugate | Thermo Fisher Scientific | Cat# A-11012, RRID: AB_2534079 |
| Goat anti-rabbit horseradish peroxidase (HRP)-conjugated IgG Secondary Antibodies | Beyotime Biotechnology | Cat# A0208 |
| Goat anti-mouse horseradish peroxidase (HRP)-conjugated IgG Secondary Antibodies | Beyotime Biotechnology | Cat# A0216 |
| Chemicals, Peptides, and Recombinant | | |
| SB290157 trifluoroacetate | MedChemExpress | Cat# HY-101502A |
| MG-132 | MedChemExpress | Cat# HY-13259 |
| JSH-23 | MedChemExpress | Cat# HY-13982 |
| TBHQ | MedChemExpress | Cat# HY-100489 |
| SC-10 | MedChemExpress | Cat# HY-100931 |
| SC79 | MedChemExpress | Cat# HY-18749 |
| Ro 67-7476 | MedChemExpress | Cat# HY-100403 |
| GSK621 | MedChemExpress | Cat# HY-100548 |
| Recombinant mouse complement component C3a protein, CF | Sino Biological | Cat# 56999-M08H |
| Me4BodipyFL-Ahx3Leu3VS (Proteasome Activity Probe) | R&D Systems | Cat# I-190-050 |
| Poly-L-Lysine | Gibco | Cat# A3890401 |
| Critical Commercial Assays | | |
| YF-594 Click-iT EdU Imaging Kits | US | Cat# C6015 |

| | | |
|---|---|---|
| | EVERBRIGHT INC. | |
| Aggresome Detection Kit | Abcam | Cat# ab139486 |
| Dual-luciferase reporter assay Kit | Promega | Cat# E1910 |
| Immunoprecipitation Kit | Beyotime Biotechnology | Cat# P2179S |
| Enzymatic ChIP Assay Kit | Beyotime Biotechnology | Cat# P2080S |
| Experimental Models: cell lines | | |
| Primary C57BL/6 mice: passage 2-5 NSCs | This paper | N/A |
| Human HEK293T | American Type Culture Collection | RRID: CVCL_0045 |
| Experimental Models: organisms/strains | | |
| Mouse: newborn male/female C57BL/6 mice (postnatal 1d) | Zhuhai BesTest Bio-Tech Co.,.Ltd. | N/A |
| oligonucleotides | | |
| Primers used for qPCR, see Table S2 | This paper | N/A |

**Table S2.** The primer sequences used in qRT-PCR assays.

| Gene | Primer sequences |
|---|---|
| *Nf-κb p65* | Forward primer (5'–3'): TCCTGTTCGAGTCTCCATGCAG<br>Reverse primer (5'–3'): GGTCTCATAGGTCCTTTTGCGC |
| *Nrf2* | Forward primer (5'–3'): CAGCATAGAGCAGGACATGGAG<br>Reverse primer (5'–3'): GAACAGCGGTAGTATCAGCCAG |
| *Pkcα* | Forward primer (5'–3'): ACAACCTGGACAGAGTGAAACTC<br>Reverse primer (5'–3'): CTTGATGGCGTACAGTTCCTCC |

| | |
|---|---|
| *Pkcβ* | Forward primer (5'–3'): CCAAGATGACGATGTGGAGTGC<br>Reverse primer (5'–3'): CTCCATCACAAAGTACAGGCGG |
| *Pkcγ* | Forward primer (5'–3'): ACGCAGCTTCACTCCACCTTTC<br>Reverse primer (5'–3'): TGGCGATTTCCGCAGCGTAGAA |
| *Gsk3β* | Forward primer (5'–3'): GAGCCACTGATTACACGTCCAG<br>Reverse primer (5'–3'): CCAACTGATCCACACCACTGTC |
| *Akt* | Forward primer (5'–3'): GGACTACTTGCACTCCGAGAAG<br>Reverse primer (5'–3'): CATAGTGGCACCGTCCTTGATC |
| *Jnk1* | Forward primer (5'–3'): CGCCTTATGTGGTGACTCGCTA<br>Reverse primer (5'–3'): TCCTGGAAAGAGGATTTTGTGGC |
| *Jnk2* | Forward primer (5'–3'): GTCAGTGGGTTGCATCATGGGA<br>Reverse primer (5'–3'): ACTCTGCGGATGGTGTTCCTAG |
| *Jnk3* | Forward primer (5'–3'): CGCTACCAGAACCTGAAGCCAA<br>Reverse primer (5'–3'): GGCGTGAGTTTGGTTCTGGAAG |
| *Erk1* | Forward primer (5'–3'): GGCTTTCTGACGGAGTATGTGG<br>Reverse primer (5'–3'): GTTGGAGAGCATCTCAGCCAGA |
| *Erk2* | Forward primer (5'–3'): TCAAGCCTTCCAACCTCCTGCT<br>Reverse primer (5'–3'): AGCTCTGTACCAACGTGTGGCT |
| *Erk5* | Forward primer (5'–3'): CAGCCTTCTACATCAGAGTCACC<br>Reverse primer (5'–3'): CCTTTGGAGTGCCAGAGAACAC |
| *P38α* | Forward primer (5'–3'): CCGAACGATACCAGAACCTGTC<br>Reverse primer (5'–3'): ACGCAACTCTCGGTAGGTCCTT |
| *Ampkα1* | Forward primer (5'–3'): GGTGTACGGAAGGCAAAATGGC<br>Reverse primer (5'–3'): CAGGATTCTTCCTTCGTACACGC |
| *Ampkα2* | Forward primer (5'–3'): CTGAAGCCAGAGAATGTGCTGC<br>Reverse primer (5'–3'): GAGATGACCTCAGGTGCTGCAT |

| | |
|---|---|
| *Perk* | Forward primer (5'–3'): CCGATGTCAGTGACAACAGCTG<br>Reverse primer (5'–3'): AAGACAACGCCAAAGCCACCAC |
| *Gapdh* | Forward primer (5'–3'): CATCACTGCCACCCAGAAGACTG<br>Reverse primer (5'–3'): ATGCCAGTGAGCTTCCCGTTCAG |
